# Integrated Bioinformatics, Environmental Epidemiologic and Genomic Approaches to Identify Environmental and Molecular Links between Endometriosis and Breast Cancer

**DOI:** 10.3390/ijms161025285

**Published:** 2015-10-23

**Authors:** Deodutta Roy, Marisa Morgan, Changwon Yoo, Alok Deoraj, Sandhya Roy, Vijay Kumar Yadav, Mohannad Garoub, Hamza Assaggaf, Mayur Doke

**Affiliations:** 1 Department of Environmental & Occupational Health, Florida International University, Miami, FL 33199, USA; E-Mails: mmorg003@fiu.edu (M.M.); adeoraj@fiu.edu (A.D.); mgaro005@fiu.edu (M.G.); hassagga@fiu.edu (H.A.); mdoke001@fiu.edu (M.D.); 2 Department of Biostatistics, Robert Stempel College of Public Health and Social Work, Florida International University, Miami, FL 33199, USA; E-Mail: cyoo@fiu.edu; 3 BMSNF Inc., Aventura, FL 33180, USA; E-Mail: roy.sandhya09@gmail.com; 4 Department of Physics, GKPD College, Karpoorigram, Samastipur 848129, India; E-Mail: sundrian9@gmail.com

**Keywords:** bioinformatics, breast cancer, bisphenol A, endocrine disruptors, endometriosis, genomics, PCBs, phthalates

## Abstract

We present a combined environmental epidemiologic, genomic, and bioinformatics approach to identify: exposure of environmental chemicals with estrogenic activity; epidemiologic association between endocrine disrupting chemical (EDC) and health effects, such as, breast cancer or endometriosis; and gene-EDC interactions and disease associations. Human exposure measurement and modeling confirmed estrogenic activity of three selected class of environmental chemicals, polychlorinated biphenyls (PCBs), bisphenols (BPs), and phthalates. Meta-analysis showed that PCBs exposure, not Bisphenol A (BPA) and phthalates, increased the summary odds ratio for breast cancer and endometriosis. Bioinformatics analysis of gene-EDC interactions and disease associations identified several hundred genes that were altered by exposure to PCBs, phthalate or BPA. EDCs-modified genes in breast neoplasms and endometriosis are part of steroid hormone signaling and inflammation pathways. All three EDCs–PCB 153, phthalates, and BPA influenced five common genes—*CYP19A1*, *EGFR*, *ESR2*, *FOS*, and *IGF1*—in breast cancer as well as in endometriosis. These genes are environmentally and estrogen responsive, altered in human breast and uterine tumors and endometriosis lesions, and part of Mitogen Activated Protein Kinase (MAPK) signaling pathways in cancer. Our findings suggest that breast cancer and endometriosis share some common environmental and molecular risk factors.

## 1. Introduction

Breast cancer and endometriosis are multifactorial complex chronic diseases with both genetic and environmental contributors. Many environmental and molecular risk factors common to breast cancer and endometriosis have received insufficient attention in molecular epidemiologic investigations because these studies have reported inconsistent results of an association with these contributors. Both breast cancer and endometriosis have in common one of the etiological factors, *i.e.*, estrogen [1]. Unopposed estrogen stimulates the growth of endometrial cells in the uterus as well as proliferation of breast cells. Tamoxifen, a hormonal therapy for breast cancer, stimulates the growth of endometrial cells and can cause endometriosis [2]. Altered endogenous estrogen is linked with an increased risk of endometriosis and breast cancer among postmenopausal women [3,4,5]. Recently, a new report by the United Nations Environment Programme (UNEP) and World Health Organization (WHO) entitled “State of the Science: Endocrine Disrupting Chemicals-2012” highlighted that approximately 800 chemicals are suspected to act as endocrine disruptors (EDs) or mimic natural hormones or disrupt hormone regulation [6,7]. Some of these EDs mimic natural or synthetic estrogen. This recent UNO report has renewed the concern by highlighting that there may be some associations between exposure to estrogen-mimicking EDs and an increased risk of breast cancer in women [6,7]. The estrogen mimicking EDs include a variety of chemicals such as pesticides, fungicides, industrial compounds, by-products of industrial processes, and chemicals used in the manufacturing of plastics. Indeed the estrogen-mimicking EDs that are persistent in the environment, highly lipophilic, and they readily bio-accumulate and magnify within the food chain [4,5,6,7]. Many of these chemicals are used in a variety of consumer products; therefore exposure to endocrine disrupting chemicals (EDCs) among the general population is widespread. Human exposure to EDCs may result from inhalation through the air, absorption through the skin, and most commonly through the ingestion of contaminated food and water [8,9]. EDCs may produce a wide range of adverse effects because of the complexity of the endocrine system with its multiple signaling pathways, feedback mechanisms and cross-talks. Although a number of experimental animal studies have shown that many chemicals have potential endocrine disrupting activities, the data, however, on their endocrine disrupting effects in humans is limited. The role of EDC’s in the etiology of some of the human cancers and reproductive health hazards has been implicated, although the linkage between these two processes is highly controversial [8]. In addition to their endocrine disrupting effects, some environmental estrogen-like chemicals produce multiple genetic and/or non-genetic hits, which may contribute to the induction of genomic instability in stem cells [4]. In the last decade, exposure to multiple EDCs such as polychlorinated biphenyls (PCBs), phthalates, and bisphenol A (BPA) have been detected in >90% of blood and urine samples collected [8,10,11,12]. PCBs have been shown to interfere with reproductive function and development in animals and humans by either increasing or blocking estrogen activity [4,5,6,7]. Adverse reproductive health effects have been established in a number of animal studies that linked PCB exposures to decreased sperm fertilizing ability in mice [13], changes in the uterine myometrium and gland formation in mice [14], and a significant dose-dependent relationship in the prevalence and severity of endometriosis in rhesus monkeys [15]. Among phthalates, di-(2-ethylhexyl) phthalate (DEHP), di-butyl phthalate (DBP) and butylbenzylphthalate (BBP) have been studied for their endocrine disrupting effects. Phthalates have been shown to produce anti-androgenic effects by suppressing testosterone and estrogen production. Exposure to high levels of phthalate have been reported to result in reproductive abnormalities in female rodents that included increased uterine and ovarian weights and malformations, delayed onset of puberty; and modified morphology of the mammary gland [16]. The majority of human exposure to BPA is via ingestion of contaminated food products [4,9]. We have shown that BPA is oxidized to bisphenol-*o*-quinone by cytochrome P450 activation system. Administration of a single dose or multiple doses of 200 mg/kg of BPA to CD1 male rats produces *in vivo* DNA adducts matching the profile of dGMP-bis-phenol-*o*-quinone. Covalent modifications in DNA by *in vivo* exposure of BPA are suspected to be a factor in the induction of endocrine toxicity [17]. In rodent females, BPA exposure has been shown to cause alterations in both development and gene expression of the mammary gland, cystic ovaries, endometrial hyperplasia, adenomyosis, leiomyomas, atypical hyperplasia, stromal polyps, ductal hyperplasia and carcinoma, a decline in fertility and fecundity, decreased wet weight of the vagina, decreased volume of the endometrial lamina propria, and an increased expression of estrogen receptor-α (ERα) and progesterone receptors [17,18,19,20,21,22,23]. Based on this body of evidence, we postulate that exposure to EDCs during early development of the breast, endometrium, and prostate may not only alter their development, but also contribute to increased susceptibility to complex chronic diseases via chemical-induced effects on stem cells.

There is a general agreement that human populations are constantly exposed to a wide variety of environmental estrogen-like chemicals. We are beginning to acknowledge endocrine disrupting effects of these chemicals in humans through experimental animal data and epidemiological studies [6,7]. Only a limited number of EDCs, such as diethylstilbestrol (DES), BPA, PCBs, phthalates and dichlorodiphenyltrichloroethane (DDT), have been studied extensively to assess the endocrine disrupting effects in experimental models and in humans. Through research on hormonal contraception, postmenopausal hormonal therapies and estrogen-receptor (ER)-based endocrine therapies, we know that estrogens are a major risk factor of both breast cancer and endometriosis [1,2,3]. The proven contribution of unopposed estrogens to the risk for breast cancer, endometriosis or endometrial neoplasia have further renewed health concerns about estrogen mimicking EDCs found in food, personal care products or as environmental contaminants. PCBs, BPA, and phthalates are the most extensively studied EDCs, and therefore, this article is focused mainly on analyzing the molecular risk factors of breast cancer and endometriosis in association with exposure of these three selected classes of EDCs–PCBs, BPA, and phthalates.

**Figure 1:**
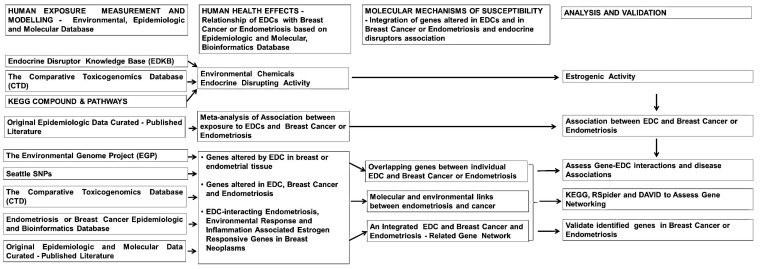
The flow chart shows the steps involved in assessing human exposure and health effects of endocrine disrupting chemicals, and identifying the molecular link between endometriosis and breast cancer based on environmental response on epidemiologic, genomics, and bioinformatics databases.

While there are studies which link EDCs–PCBs, BPA, and phthalate exposure to an increased risk of breast cancer or endometriosis, there have also been inconsistent study findings with reports of no association. In this study, we used a combined environmental epidemiologic, genomic, and bioinformatics approach to understand the relationship between EDCs and risk of developing estrogen-dependent breast cancer and endometriosis, by examining interactions between genes, diseases and these three selected classes of EDCs. We also evaluated the possibility that “estrogen mimicking endocrine disruptor responsive genes” are potentially associated with systemic changes in the etiology of breast cancer and endometriosis. Here we used a comprehensive approach to integrate bioinformatics, genomics, environmental and epidemiologic data to identify (1) genes that interact with three classes of EDCs; and (2) molecular pathways that are potentially influenced by EDC exposures that potentially links with the development of breast cancer and/or endometriosis (Figure 1). The first and second steps in our method included modeling to assess estrogenicity of environmental chemicals to identify the potential for endocrine disruption and assessing association between EDC exposure and diseases, respectively. The third step included identifying responsive genes to EDC exposures using the Comparative Toxicology Database (CTD), Environmental Genome Project and Kyoto Encyclopedia of Genes and Genomes (KEGG). These EDC responsive genes were then compared to a curated list of genes in breast cancer and endometriosis. This comparison produced a list of genes responsive to the environment and important to breast cancer and endometriosis that was then further analyzed using gene networking tools such as RSpider, Cytoscape, and DAVID. Using this comprehensive approach to integrate bioinformatics, genomics, environmental and epidemiologic data, we were able to identify environmentally responsive genes that are potentially involved in interactions with EDCs and may be significant for the development of breast cancer and endometriosis. Potential gene-EDCs interactions may help generate novel hypotheses to further evaluate the biological-based mechanisms and better understand the significant impact that EDC exposures have on the etiology of breast cancer and endometriosis.

## 2. Results

### 2.1. Human Exposure Measurement and Modeling

First, we searched the EDCs-gene associations in CTD which revealed that the most common studied EDCs for gene interaction were bisphenol A, bisphenol A-glycidyl methacrylate, dibutyl phthalate, diethylhexyl Phthalate; and PCB congeners—3,4,3′,4′-tetrachlorobiphenyl (77), 2′,3,3′,4′,5-pentachloro-4-hydroxybiphenyl (4′-OH-PCB-86), 3,4,5,3′,4′-pentachlorobiphenyl (126), 2,3,3′,4,4′,5-hexachlorobiphenyl (153), and 2,2′,3,4,4′,5,5′-heptachlorobiphenyl (180). We used these EDCs to assess their estrogenic activity. A number of exposure models have been proposed for EDCs. We mapped these chemicals onto the KEGG endocrine disrupting compound, the KEGG pathway and metabolic pathways, particularly synthetic and degradation pathways of EDCs, CTD based analysis of estrogen receptor signaling pathway genes, and Endocrine Disruptor Knowledge Base (EDKB) computational models. These genomic web based tools predicted estrogenic activity of all EDCs, except bisphenol a-glycidyl methacrylate and was consistent with the previous reports [4,5,6,7]. Bisphenol a-glycidyl methacrylate was not active.

### 2.2. Meta-Analysis of Association between Exposure to EDCs and Risk of Breast Cancer and Endometriosis

Here we reviewed and meta-analyzed environmental epidemiologic evidence for the risk of breast cancer with exposure to EDCs-PCB, phthalates, and BPA.

#### 2.2.1. PCBs and Breast Cancer

Of the 125 publications we identified in our search, we based our meta-analysis on evidence from 23 selected publications of epidemiological studies which we categorized by outcome: breast cancer and endometriosis. The measure of exposure varied slightly between studies. PCB concentrations were measured in serum (*n* = 154) or plasma (*n* = 2), phthalate concentrations were measured in urine (*n* = 3) or plasma (*n* = 1), and BPA concentrations were measured in urine (*n* = 1) or blood (*n* = 1). All of the selected studies calculated unadjusted and/or adjusted arithmetic means, geometric means, medians, or mean TEQ/kg values to assess and compare EDC exposure among cases and controls. Furthermore, all of the studies estimated ORs and 95% CIs for breast cancer and endometriosis using unadjusted and/or adjusted logistic regression models. We identified twelve epidemiologic studies related to PCB, phthalate, or BPA exposure and breast cancer. Ten of the twelve studies assessed the relationship between PCB exposure and breast cancer [24,25,26,27,28,29,30,31,32,33,34], one study assessed the relationship between phthalate exposure and breast cancer [35], and one study assessed the relationship between BPA exposure and breast cancer [36]. All twelve of the identified studies were case-control studies. In all of the studies, cases had histologically confirmed breast cancer and controls had no history of breast cancer diagnosis. In the majority of the studies, controls were matched on age and residence.

All ten of the studies that addressed the relationship between PCB exposure and breast cancer presented individual PCB congener results as well as a measure of total PCBs, the sum of individual congeners. To summarize the main results between PCB exposure and breast cancer, lipid adjusted arithmetic means or geometric means of total PCB exposure were provided for seven studies [24,27,28,31,33,34,37], median lipid adjusted PCB levels were provided in one study [26], and mean TEQ/kg of lipids was provided in one study [30]. Furthermore, all studies estimated ORs and 95% CIs for breast cancer using adjusted and/or unadjusted logistic regression models with eight of the ten studies using tertiles, quartiles, or quintiles to compare highest *versus* lowest exposure categories.

Three of the ten PCB case-control studies failed to find any associations between exposure to total PCBs and breast cancer risk [28,29,34], while two of the ten PCB studies found an inverse association between total PCB levels and breast cancer [30,32]. The largest case-control study conducted by Gammon *et al.* [27] consisted of 646 newly diagnosed breast cancer cases and 429 matched controls failed to find any association between PCB exposure and breast cancer risk when comparing the highest quintile of serum Peak-4 (nos. 118, 153, 138, and 180) PCB levels to the lowest quintile (OR = 0.83, 95% CI 0.54–1.29). Gatto *et al.* [28] did not find any associations with breast cancer when comparing the highest *vs.* lowest quintiles of mean total PCB levels in 355 cases and 327 controls (OR = 1.01, 95% CI 0.63–1.63) and Wolff *et al.* [33] did not find any associations with breast cancer when comparing the highest *vs.* lowest quartiles of serum total PCB levels in 110 cases and 213 controls. Itoh *et al.* [29] found a decreased risk of breast cancer when comparing the highest quartile of median total PCB levels to the lowest quartile (OR = 0.33, 95% CI 0.14–0.78) and Pavuk *et al.* [31] found higher serum PCB levels to be inversely associated with breast cancer in total PCBs (OR = 0.42, 95% CI 0.10–1.82) and in three sub-groups of PCBS: estrogenic, anti-estrogenic/dioxin-like, and phenobarbital-type.

Five of the PCB case-control studies found significant associations between breast cancer and exposure to individual PCB congeners, total PCBs, or specific sub-groups of PCBs [24,25]. Charlier *et al.* [24] measured mean levels of seven PCB congeners in 60 breast cancer cases and 60 healthy controls. They found that total PCBs to be significantly different (*p* = 0.012) between cases (7.08 ppb) and controls (5.10 ppb) and significantly higher serum levels of PCB 153 in breast cancer cases when compared to controls (1.63 *vs.* 0.63 ppb, *p* < 0.0001). The OR of breast cancer for PCB 153 was 1.8 (95% CI 1.4–2.5). In a nested, matched case-control study of 112 cases and controls, Cohn *et al.* [25] did not find any associations for total PCBs or PCB groupings, however, a significant association was found for PCB 203 when comparing the highest *vs.* lowest quartiles of exposure (OR = 6.3, 95% CI 1.9–21.7). In a matched case-control study of 314 cases and 523 controls, Demers *et al.* [26] found breast cancer risk significantly associated with the sum of mono-ortho congeners (nos. 105, 118, 156) (OR = 2.02, 95% CI 1.24–3.28), PCB 118 (OR = 1.60, 95% CI 1.01–2.53) and PCB 156 (OR = 1.80, 95% CI 1.11–2.94) when comparing the fourth *vs.* first quartiles. In a population based case-control study with sub-groups of African-American women and white women, Millikan *et al.* [35] did not find any associations with total PCBs and breast cancer among all participants (OR = 1.09, 95% CI 0.79–1.52) or white women (OR = 1.03, 95% CI 0.68–1.56), but did find a slightly elevated risk for African-American women (OR = 1.74, 95% CI 1.00–3.01). Recio-Vega *et al.* [32] found the GM of total PCBs to be significantly higher in cases than controls (5.26 *vs.* 3.33 ppb) (OR = 1.09, 95% CI 1.01–1.14) as well as an increased risk of breast cancer among PCBs grouped by structure-activity relationships and eight individual PCB congeners (nos. 118, 128, 138, 170, 180, 195, 206, and 209).

**Figure 2:**
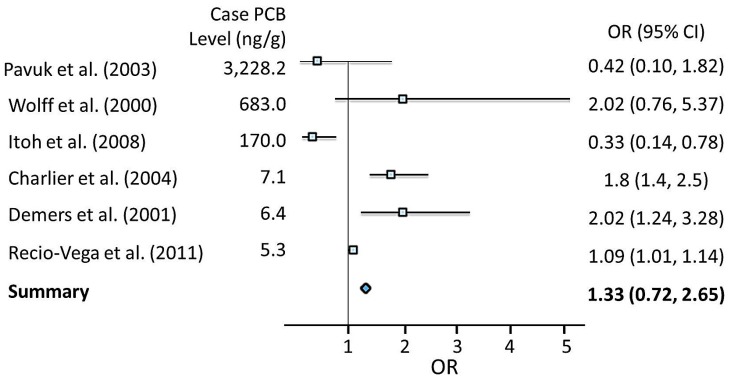
Forest plot of Epidemiological studies of the association between exposure to PCBs and risk of breast cancer.

Since the relationship between PCB exposure and breast cancer in ten epidemiologic studies was inconsistent or conflicting, risk estimates of PCBs on breast cancer from six case control studies were extracted and summarized using meta-analytic methods. Combining six studies of exposure to PCBs produced a summary risk estimate of 1.33 (95% CI: 0.72–2.65) (Table 1; Figure 2). However, PCB exposures were found to be associated with development of breast cancer as a meta-analysis of six studies produced an increased summary of OR risk of 1.33, this was not statistically significant.

**Table 1:** Epidemiological studies of the association between exposure to PCBs and risk of breast cancer.

Reference, Location	Study Design	Study Population	Measurement of Exposure	Outcomes	Results	Comments	Confounders
Charlier *et al.* [24], Belgium	Case-control study	60 cases, 60 age matched healthy controls	7 PCBs from serum, Total PCBs.	Mean Total PCB levels (ppb = ng/g) Cases: 7.08; Controls: 5.10; Logistic Regression (OR, 95% CI).	Total PCBs significantly different in cases than controls (*p* = 0.012). High concentrations of PCB153 significantly associated with an increased risk of BC (OR = 1.8, 95% CI: 1.4–2.5).	Cases diagnosed with breast cancer and undergoing a surgical intervention. Controls free of BC at age of diagnosis.	Adjustments made for age, menopausal status, number of full-term pregnancies, lactation, use of HRT, and family history of BC.
Demers *et al.* [26], Canada	Case-control study	314 cases, 523 controls; matched by age and residence	14 PCB congeners measured in plasma (μg/kg of plasma lipids). TEQ/kg of lipids for sum of mono-ortho congeners (nos. 105, 118, 156).	Mean TEQ ng/g of lipids: Cases: 6.4; Controls: 5.8; Logistic Regression (OR, 95% CI); Quartiles.	Mean total of mono-ortho congeners significantly higher in cases than controls (*p* = 0.005). Significant associations between breast cancer risk and PCB 156, 118, & mono ortho congeners. In 4th *vs.* 1st quartiles. (OR = 2.02, 95% CI: 1.24–3.28).	Cases: histologically confirmed infiltrating primary BC. Controls: no history of BC diagnosis.	Adjusted for age, residence, BMI, history of benign breast disease, breastfeeding duration.
Pavuk *et al.* [31], USA	Case-control study	24 cases, 88 controls	Total PCBs from serum (*n* = 15); Groups of PCBs: (1) estrogenic; (2) anti-estrogenic, dioxin-like; (3) phenobarbital-type.	GMs Total PCBs (ng/g of lipid): Cases: 3228.2; Controls: 2885.8. Logistic Regression (OR, 95% CI); Tertiles.	Higher serum levels of total PCBs (OR = 0.42, 95% CI 0.10–1.82) inversely associated with BC. Groups 1, 2, & 3 also inversely associated.	Cases: histologically confirmed invasive BC. Controls: identified through random sampling of primary care physicians.	Adjusted for age, age at menarche, education, alcohol consumption, smoking.
Recio-Vega *et al.* [32], Mexico	Case-control study	70 cases, 70 controls	Individual and total PCBs from serum (*n* = 20); 5 PCB groups by structure-activity relationships.	GM Total PCB levels (ppb): Cases: 5.26; Controls: 3.33. Logistic Regression (OR, 95% CI).	Total PCBs significantly higher among cases than controls (OR = 1.09, 95% CI 1.01–1.14). Risk of BC positively associated with 8 PCB congeners: 118, 128, 138, 170, 180, 195, 206, and 209.	Cases: first diagnosis of BC by biopsy. Controls: negative biopsies from same hospitals and geographic area.	Adjusted for age, age at menarche, lactation, menopause status, BMI.
Wolff *et al.* [33], USA	Prospective case-control study	148 cases, 295 individually matched controls	Total PCBs from serum.	GM Total PCBs (ng/g of lipids): Cases: 683; Controls: 663. Logistic Regression (OR, 95% CI); Quartiles.	GM Total PCB levels not significantly different. No association between PCB exposure and BC (OR = 2.02; 95% CI 0.76–5.37).	BC cases identified through active follow-up of the NYU Women’s Health Study Cohort. Controls selected at random from cohort who were alive and free of disease at the time of case diagnosis.	Adjusted for age at menarche, # of full-term pregnancies, age at first birth, family history of BC, lifetime history of lactation, BMI, menopausal status at time of blood donation.
Itoh *et al.* [29], Japan	Matched case-control study	403 pairs; matched by age (3 years) and residence	Total PCBs from serum (Sum of 41 PCB peaks).	Median Total lipid-adjusted PCBs (ng/g): Cases: 170; Controls: 180. Logistic Regression (OR, 95% CI), Quartiles.	Total PCBs associated with a decreased risk of BC. (OR = 0.33, 95% CI: 0.14–0.78, *p*-value 0.008); highest *vs.* lowest quartile.	Cases: histologically confirmed invasive BC. Controls: selected from medical checkup examinees, no BC diagnosis.	Adjusted for lipids, BMI, menopausal status & age, smoking, fish & veg consumption, family history, parity, age at first childbirth, age at menarche, history of BC screening

#### 2.2.2. Bisphenol A or Phthalate and Breast Cancer

No meta-analysis was performed on exposure to BPA or phthalates, because only one study for each chemical fit the criteria. Lopez-Carillo *et al.* [30] found urinary concentrations of monoethyl phthalate (MEP) to be significantly higher in cases than controls when comparing the highest *vs.* lowest tertile of exposure (169.58 *vs.* 106.78 μg/g creatinine). The OR of breast cancer risk in the highest tertile of urinary MEP, compared with the lowest tertile, was 2.20 (95% CI 1.33–3.63) and became higher when estimated for premenopausal women (OR = 4.13, 95% CI 1.60–10.7). On the contrary, significant negative associations were found for urinary concentrations of monobenzyl phthalate (MBzP) (OR = 0.46, 95% CI 0.27–0.79) and mono (3-carboxypropyl) phthalate (MCPP) (OR = 0.44, 95% CI 0.24–0.80). In a matched case-control study, Yang *et al.* [34] measured median blood BPA levels in 70 cases and 80 controls. Median BPA levels were higher in cases than controls (0.61 *vs.* 0.03 μg/L), however, the differences were not found to be statistically significant (*p* = 0.42).

### 2.3. PCBs–Breast Cancer–Gene Association

**Figure 3:**
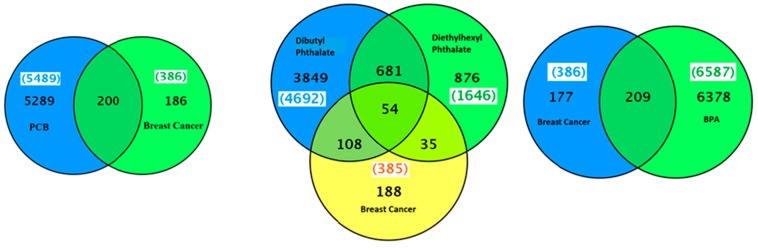
A Venn diagram of list of genes common between breast neoplasms and PCBs, phthalates or bisphenol A.

The CTD search revealed that besides PCBs, the five most common PCB congeners studied for gene interaction were 3,4,3′,4′-tetrachlorobiphenyl (77), 2′,3,3′,4′,5-pentachloro-4-hydroxybiphenyl (4′-OH-PCB-86), 3,4,5,3′,4′-pentachlorobiphenyl (126), 2,3,3′,4,4′,5-hexachlorobiphenyl (153), and 2,2′,3,4,4′,5,5′-heptachlorobiphenyl (180) (Table 2). There were 5289 genes related to PCB family of chemicals and 386 genes related to breast cancer (Figure 3). The common genes between PCBs and breast cancer were 200. The top interacting genes with PCBs as a chemical class were *CYP1A1*, *AHR*, *CYP1A2*, *AR*, *CYP1A*, *CYP1B1*, *VCAM1*, *MAPK1*, *MAPK3*, and *PTGS2*. The top interacting genes with PCBs in breast neoplasms were *AR*, *CYP1A1*, *CYP1B1*, *ESR1*, *ESR2*, *PTGS2*, and *RAF1*. Out of a total 200 genes interactions observed with individual PCBs, the interaction of genes *AR*, *BAX*, *CYP1A1*, *CYP1B1*, *KDR*, *PARP1*, *PTGS2*, and *RAF1* was common with tetrachloride, pentachloride, and hexachloride biphenyls in beast neoplasms (Table 2). *CYP1A1*, *AHR*, *AR*, *CYP1A*, *CYP1B1* and *PTGS2* genes are common in both PCB-gene and PCB-gene-breast cancer groups. Interactions among these genes are shown in Figure S1. Enrichment pathway analysis revealed that these genes are part of: (1) pathways in cancer (KEGG: 05200); (2) signal transduction (REACT: 111102); (3) mTOR signaling pathway (KEGG: 04150); (4) focal adhesion (KEGG: 04510); (5) VEGF signaling pathway (KEGG: 04370); and (6) ErbB signaling pathway (Table 3).

### 2.4. BPA, Phthalate and Breast Cancer–Gene Association

There were 6365 genes associated with the chemical BPA. There were 385 genes known to be associated with breast cancer. There were 209 genes in common between BPA and breast cancer (Figure 2). There were 5754 genes associated with phthalate chemical class and 385 genes associated to breast cancer (Figure 2). The common genes shared between dibutyl phthalate and breast cancer; and diethylhexyl phthalate and breast cancer were 162 and 89, respectively. Identification of the common genes with breast cancer and both dibutyl phthalate and diethylhexyl phthalate further revealed that there were 54 common genes between dibutyl phthalate and diethylhexyl phthalate and breast cancer as shown in Table 2 Interactions among these genes are shown in Figure 3. Enrichment pathway analysis revealed that some of these genes are part of: (1) pathways in cancer (KEGG: 05200); (2) signal transduction (REACT: 111102); and (3) MAPK signaling pathway (KEGG: 04150) (Table 3).

**Table 2:** Genes interacting with polychlorinated biphenyls in breast neoplasms.

IUPAC Name (Congener Number)	Interacting Genes
Polychlorinated biphenyls	65 genes: *ACHE* | *AFP* | *AGR2* | *AHR* | *AKAP12* | *AKT1* | *ANGPTL4* | *APOBEC3B* | *AR* | *ARAF* | *AREG* | *AURKA* | *BCHE* | *BIRC5* | *CDKN1B* | *CENPF* | *CLDN4* | *COMT* | *CXCL12* | *CXCL2* | *CYP17A1* | *CYP19A1* | *CYP1A1* | *CYP1B1* | *CYP2B1* | *CYP3A4* | *DNMT1* | *DNMT3A* | *DNMT3B* | *ESR1* | *ESR2* | *FOS* | *GPI* | *GPNMB* | *H2AFX* | *HEY1* | *HMOX1* | *HP* | *IFNG* | *IL6* | *JUN* | *KRAS* | *MKI67* | *MMP3* | *NCOA3* | *NQO1* | *PPARGC1B* | *PTGS2* | *RAD51* | *RAD51C* | *RAD54L* | *RAF1* | *RPS8* | *SOD2* | *SPP1* | *STC2* | *STMN1* | *TGM2* | *THBS1* | *THEMIS2* | *TNF* | *TOP2A* | *TYMS* | *UBE2C*
2,4,4′-Trichlorobiphenyl (28)	11 genes: *AR* | *CYP1A1* | *CYP1B1* | *CYP2B1* | *ESR1* | *ESR2* | *HIF1A* | *KDR* | *PTGS2* | *RAF1* | *TP53*
2,4′,5-Trichlorobiphenyl (31)	3 genes: *AR* | *ESR1* | *ESR2*
2,5,2′,5′-Tetrachlorobiphenyl (55)	13 genes: *ACHE* | *AHR* | *AKT2* | *APC2* | *AR* | *CYP1A1* | *CYP2B1* | *EPB41L3* | *IGF1R* | *MMP2* | *PARP1* | *PLA2G4A* | *ZEB2*
3,4,3′,4′-Tetrachlorobiphenyl (77)	27 genes: *AHR* | *AR* | *BAX* | *CAV1* | *CCNE1* | *CYP1A1* | *CYP1B1* | *ESR1* | *ESR2* | *GPI* | *GPX1* | *HADHB* | *HIF1A* | *HNRNPK* | *IL1B* | *IL6* | *KDR* | *MRPL19* | *NDRG1* | *NOS3* | *NQO1* | *PARP1* | *PER3* | *PTGS2* | *RAF1* | *RELA* | *TNF*
2′,3,3′,4′,5-Pentachloro-4-hydroxybiphenyl (4′-OH-PCB-86; 4-hydroxy-2,2′,3′,4′,5′-pentachlorobiphenyl)	75 genes: *ACVR1* | *AFP* | *APC2* | *ARAF* | *ATM* | *BIRC5* | *BMPR2* | *CASP8* | *CAV1* | *CD40* | *CHEK2* | *CSF1* | *CSF3* | *CST6* | *CXCL12* | *CXCL2* | *CYP17A1* | *CYP1A1* | *DAP3* | *DDIT3* | *DNMT3A* | *DNMT3B* | *DPYD* | *EEF2* | *EFNA1* | *EGF* | *ERBB2* | *F3* | *FABP7* | *FGFR2* | *FHL2* | *FKBPL* | *GDF10* | *HIF1A* | *HRG* | *IL24* | *JAG1* | *JAG2* | *JUN* | *LDHB* | *LEPR* | *LPAR1* | *MFGE8* | *MME* | *MMP2* | *MMP3* | *MMP9* | *MRPL13* | *MTDH* | *MTHFR* | *MTR* | *NDRG1* | *NOTCH3* | *NOTCH4* | *NRCAM* | *NUDT2* | *OCLN* | *PARP1* | *PDE2A* | *PDGFA* | *PHB* | *PPARGC1B* | *PTPRD* | *RGS2* | *SLC28A1* | *STAT3* | *SYNE1* | *SYNJ2* | *TFRC* | *THBS1* | *THEMIS2* | *TOP2A* | *VEGFC* | *VPS39* | *ZEB1*
2,2′,4,6,6′-Pentachlorobiphenyl (104)	9 genes: *AKT1* | *AR* | *CXCL8* | *EGFR* | *FOS* | *JUN* | *MMP3* | *OCLN* | *SRC*
2,3,3′,4,4′-Pentachlorobiphenyl (105)	4 genes: *AHR* | *AR* | *CYP1A1* | *CYP2B1*
2,3′,4,4′,5-Pentachlorobiphenyl (107)	10 genes: *AHR* | *AR* | *CASP7* | *CYP1A1* | *CYP1B1* | *CYP2B1* | *HIF1A* | *KDR* | *OCLN* | *PTGS2*
2,3,4,4′,5-Pentachlorobiphenyl (114)	2 genes: AHR | CYP1A1
2,3′,4,4′,5-Pentachlorobiphenyl (118)	10 genes: *AHR* | *AR* | *CASP7* | *CYP1A1* | *CYP1B1* | *CYP2B1* | *HIF1A* | *KDR* | *OCLN* | *PTGS2*
3,4,5,3′,4′-Pentachlorobiphenyl (126)	77 genes: *ACVR1* | *ADAMTS1* | *AFP* | *AHR* | *AKAP12* | *AKT1* | *APRT* | *AR* | *AREG* | *BARD1* | *BAX* | *BCL2* | *BMP4* | *BRCA1* | *CADM1* | *CAV1* | *CCL20* | *CCND1* | *CD74* | *CXCL12* | *CXCL8* | *CYP17A1* | *CYP19A1* | *CYP1A1* | *CYP1B1* | *CYP2B1* | *EGF* | *ESR1* | *F3* | *FASN* | *FGFR2* | *FHL2* | *FST* | *GPNMB* | *HES1* | *HIF1A* | *HMOX1* | *ICAM5* | *IGF1* | *IGF1R* | *IGFBP5* | *IGFBP7* | *IL1B* | *IL6* | *JUN* | *KDR* | *LDHAL6B* | *LPAR1* | *MME* | *MMP9* | *NOS2* | *NOS3* | *NOTCH1* | *NQO1* | *NRG1* | *OCLN* | *PAK1* | *PARP1* | *PDGFA* | *PER3* | *PHGDH* | *PLA2G4A* | *PTGS2* | *PTPRD* | *RAF1* | *SERPINB2* | *SLC2A5* | *SLC5A5* | *SNAI1* | *SPP1* | *STC2* | *SYNJ2* | *TBX3* | *TGM2* | *TNF* | *TP53* | *VEGFC*
2,3,4,2′,3′,4′-Hexachlorobiphenyl (128)	2 genes: *AHR* | *AR*
2,3,3′,4,4′,5-Hexachlorobiphenyl (129)	5 genes: *AHR* | *AR* | *CYP1A1* | *CYP1B1* | *ESR1*
2,2′,3′,4,4′,5-Hexachlorobiphenyl (137)	27 genes: *ACHE* | *AHR* | *AKT2* | *APC2* | *AR* | *BRCA1* | *CCT5* | *CENPF* | *CFL1* | *CYP1A1* | *CYP1B1* | *CYP2B1* | *EEF2* | *EGF* | *ENO1* | *FBL* | *FOS* | *HIF1A* | *HSP90AA1* | *IGF1* | *KDR* | *MMP2* | *NOTCH2* | *NRG1* | *PTGS2* | *STARD8* | *ZEB2*
2,3,6,2′,3′,6′-Hexachlorobiphenyl (136)	2 genes: *AR* | *CYP2B1*
2,4,5,2′,4′,5′-Hexachlorobiphenyl (153)	51 genes: *AHR* | *AKAP12* | *AR* | *BAX* | *BCL2* | *BRCA1* | *CASP8* | *CCND1* | *CDH1* | *CTNNB1* | *CXCL12* | *CYP19A1* | *CYP1A1* | *CYP1B1* | *CYP2B1* | *CYP3A4* | *EGFR* | *ESR1* | *ESR2* | *FASN* | *FOS* | *GPX1* | *GPX2* | *HIF1A* | *HSPA1B* | *IFNG* | *IGF1* | *IL1B* | *JUN* | *KDR* | *MAP3K1* | *MEIS1* | *NDRG1* | *NOTCH1* | *NQO1* | *NRG1* | *OCLN* | *PARP1* | *PTGS2* | *PTPRD* | *RAF1* | *SPP1* | *SRC* | *STAT5A* | *STMN1* | *TFPI2* | *TNF* | *TNFSF10* | *TP53* | *TUBB3* | XRCC3
3,4,5,3′,4′,5′-Hexachlorobiphenyl	7 genes: *AHR* | *BAX* | *CYP1A1* | *CYP1B1* | *HIF1A* | *KDR* | *PTGS2*
2,2′,3,4,4′,5,5′-Heptachlorobiphenyl (180)	19 genes: *ABL1* | *AHR* | *AR* | *BAX* | *BCL2* | *BRCA1* | *CYP1A1* | *CYP1B1* | *CYP2B1* | *FOS* | *HIF1A* | *IGF1* | *KDR* | *MMP2* | *MMP9* | *NOTCH1* | *PTGS2* | *TP53* | *ZEB2*
17β Estradiol	255 genes: *ABCG2* | *ACHE* | *ADAMTS1* | *AFP* | *AGR2* | *AHR* | *AKAP12* | *AKT1* | *AKT2* | *ALDOA* | *APOBEC3B* | *AR* | *ARAF* | *AREG* | *ARHGDIA* | *ARTN* | *ATM* | *ATP7B* | *AURKA* | *BARD1* | *BAX* | *BCAR3* | *BCHE* | *BCL2* | *BIRC5* | *BMP2* | *BMP4* | *BMPR2* | *BRCA1* | *BRCA2* | *C10ORF10* | *CADM1* | *CASP7* | *CASP8* | *CAV1* | *CCL20* | *CCND1* | *CCNE1* | *CD109* | *CD40* | *CDA* | *CDH1* | *CDH5* | *CDKN1B* | *CENPF* | *CFL1* | *CHEK2* | *CLDN1* | *CLDN4* | *COL7A1* | *COMT* | *CSF1* | *CSF1R* | *CSF3* | *CST6* | *CTNNB1* | *CXCL12* | *CXCL2* | *CXCL3* | *CXCL8* | *CYP17A1* | *CYP19A1* | *CYP1A1* | *CYP1B1* | *CYP2B1* | *CYP3A4* | *DDIT3* | *DKK1* | *DNMT1* | *DNMT3A* | *E2F1* | *EDNRB* | *EFEMP1* | *EFNA1* | *EGF* | *EGFR* | *ELK3* | *ENO1* | *EPHB4* | *EPOR* | *ERBB2* | *ESR1* | *ESR2* | *ESRRA* | *ETS2* | *ETV4* | *EVL* | *F3* | *FASN* | *FBL* | *FGF10* | *FGFR1* | *FGFR2* | *FHL2* | *FKBPL* | *FOS* | *FOXA1* | *FOXM1* | *FOXP3* | *FST* | *GDF10* | *GPNMB* | *GPX1* | *GPX2* | *GPX4* | *GRB7* | *H2AFX* | *HADHB* | *HES1* | *HEY1* | *HEY2* | *HIF1A* | *HIST1H1C* | *HIST1H2BC* | HIST1H2BK | *HMMR* | *HMOX1* | *HP* | *HPSE* | *HRAS* | *HRG* | *HSP90AA1* | *HSPA1B* | *IFNG* | *IGBP1* | *IGF1* | *IGF1R* | *IGFBP5* | *IL1B* | *IL24* | *IL6* | *JAG1* | *JUN* | *KCNH1* | *KDR* | *KIT* | *KRAS* | *KRT18* | *KRT8* | *LDHB* | *LEP* | *LEPR* | *LOXL2* | *LPAR1* | *LSP1* | *MAL* | *MAP3K1* | *MDM4* | *MIF* | *MIR10B* | *MIR146A* | *MIR200B* | *MIR222* | *MKI67* | *MME* | *MMP1* | *MMP2* | *MMP3* | *MMP9* | *MTR* | *NAT2* | *NCOA1* | *NCOA2* | *NCOA3* | *NCOR1* | *NDRG1* | *NFKBIA* | *NOS2* | *NOS3* | *NOTCH1* | *NOTCH2* | *NQO1* | *NQO2* | *NR2F1* | *NRG1* | *NRIP1* | *NUDT2* | *PAEP* | *PAK1* | *PARP1* | *PDGFA* | *PGR* | *PHB* | *PHGDH* | *PIK3CA* | *PIM1* | *PLA2G4A* | *PPARGC1B* | *PPM1D* | *PTEN* | *PTGS1* | *PTGS2* | *PTHLH* | *RAD51* | *RAD51C* | *RAF1* | *RARB* | *RB1* | *RBM3* | *RELA* | *RGS2* | *RPL31* | *RPS4X* | *RPS6* | *RPS7* | *RRAD* | *SERPINB2* | *SERPINB5* | *SFRP1* | *SFRP2* | *SLC2A1* | *SLC2A2* | *SLC2A5* | *SLC39A6* | *SLC5A5* | *SNAI1* | *SNAI2* | *SOD2* | *SPP1* | *SRC* | *STAT3* | *STAT5A* | *STC2* | *STMN1* | *SYNE1* | *SYNJ2* | *TANK* | *TBX3* | *TCL1B* | *TERT* | *TFAP2A* | *TFPI2* | *TFRC* | *TGM2* | *THBS1* | *THEMIS2* | *TLE3* | *TNF* | *TNFSF10* | *TNIP1* | *TOP2A* | *TOX3* | *TP53* | *TRERF1* | *TRP53* | *TUBB3* | *TYMS* | *UBE2C* | *VPS39* | *WNT10B* | *WT1* | *ZEB1* | *ZEB2* | *ZNF365* | *ZNF366*
Diethyl phthalate	9 genes: *AFP* | *AHR* | *AR* | *CXCL8* | *CYP17A1* | *CYP1B1* | *ESR1* | *ESR2* | *IFNB1*
Dibutyl phthalate and diethylhexyl phthalate	54 Common genes: *ABCG2* | *AHR* | *AKT1* | *ALDOA* | *AR* | *BCL2* | *BMP2* | *BMP4* | *CADM1* | *CASP7* | *CCND1* | *CD40* | *CTNNB1* | *CYP17A1* | *CYP19A1* | *CYP1A1* | *CYP1B1* | *CYP3A4* | *DNMT1* | *DNMT3A* | *DNMT3B* | *EDNRB* | *EEF2* | *EGFR* | *ESR1* | *ESR2* | *ESRRA* | *F3* | *FASN* | *FOS* | *GPX1* | *HADHB* | *HSP90AA1* | *IGF1* | *IGFBP7* | *JUN* | *LOXL2* | *MMP2* | *MMP9* | *NDRG1* | *NFKBIA* | *NOTCH2* | *OCLN* | *PER3* | *PIK3CA* | *PTPRD* | *RPL31* | *RPS4X* | *SOD2* | *THBS1* | *TNF* | *TUBB3* | *WNT10B* | *YBX1*
Bisphenol A	209 genes: *ABCG2* | *ABL1* | *ACHE* | *AFP* | *AGR2* | *AHR* | *AKAP12* | *AKT1*| *ALDOA* | *APOBEC3B* | *AR* | *ARAF* | *AREG* | *ARHGDIA* | *AURKA* | *BAG1* |*BARD1* | *BAX* | *BCAR3* | *BCL2* | *BCL2A1* | *BIRC5* | *BMP4* | *BRCA1* | *BRCA2* |*CASP7* | *CASP8* | *CAV1* | *CCND1* | *CCNE1* | *CCT5* | *CDH1* | *CDH5* | *CDKN1B* |*CENPF* | *CFL1* | *CHEK2* | *CLDN4* | *CMC2* | *COTL1* | *CSF2* | *CTNNB1* | *CUL5* |*CXCL12* | *CXCL3* | *CYP17A1* | *CYP19A1* | *CYP1A1* | *CYP1B1* | *CYP2D6* |*CYP3A4* | *DAP3* | *DDIT3* | *DNMT1* | *DNMT3A* | *DNMT3B* | *DSC3* | *E2F1* | *EEF2* |*EGF* | *EGFR* | *ENO1* | *ERBB2* | *ESR1* | *ESR2* | *ESRRA* | *ETS2* | *EVL* | *EZH2* | *FASN*| *FBL* | *FGFR1* | *FGFR2* | *FHL2* | *FOS* | *FOXM1* | *FST* | *GDF10* | *H2AFX* | *HADHB* |*HES1* | *HEY2* | *HIC1* | *HMMR* | *HMOX1* | *HNRNPL* | *HNRNPR* | *HP* | *HRAS* |*HSP90AA1* | *HSPA1B* | *IFNB1* | *IFNG* | *IGBP1* | *IGF1* | *IGF1R* | *IGFBP5* | *IL1B* |*IL6* | *JAG1* | *JAG2* | *JUN* | *KDR* | *KIT* | *KRAS* | *KRT8* | *LEP* | *LEPR* | *LLGL1* |*LPAR1* | *MAL* | *MAP3K1* | *MDM4* | *MEIS1* | *MFGE8* | *MIF* | *MIR146A* | *MIR200B* |*MIR222* | *MIR29A* | *MIR342* | *MKI67* | *MME* | *MMP1* | *MMP2* | *MMP9* | *MRPL13* |*MRPL19* | *MRPS22* | *MTR* | *NAT2* | *NCOA1* | *NCOA2* | *NCOA3* | *NCOR1* | *NDRG1*| *NOS2* | *NOS3* | *NOTCH1* | *NOTCH2* | *NOTCH3* | *NQO1* | *NRCAM* | *NRIP1* |*NUDT2* | *OCLN* | *PAK1* | *PARP1* | *PDGFA* | *PER3* | *PGR* | *PHB* | *PHGDH* | *PIM1* |*PIN1* | *PLA2G4A* | *PTEN* | *PTGS1* | *PTGS2* | *RAD51* | *RAD51B* | *RAD51C* |*RAD54L* | *RB1* | *RELA* | *RGS2* | *RIBC2* | *RPS6* | *RPS6KB2* | *RPS7* | *RXRB* |*SERPINB5* | *SFRP1* | *SFRP2* | *SHMT1* | *SIRT1* | *SLC22A18* | *SLC2A1* | *SLC2A2* |*SLC5A5* | *SNAI2* | *SOD2* | *SPP1* | *SRC* | *STAT3* | *STAT5A* | *STC2* | *STMN1* |*SYNE1* | *TANK* | *TBX3* | *TERT* | *TFAP2A* | *TFPI2* | *TGM2* | *THBS1* | *TNF* | *TNFSF10*| *TNIP1* | *TOP2A* | *TP53* | *TYMS* | *UBE2C* | *UMPS* | *WNT10B* | *WT1* | *WWOX* |*XRCC3* | *YBX1*

**Table 3:** KEGG enrichment pathways for common genes between EDCs, breast cancer and endometriosis.

Pathways	Pathway ID	Gene Association	Number of Associated Genes
Steroid hormone biosynthesis	KEGG:00140	*CYP19A1*	1
Metabolic pathways	KEGG:01100	*CYP19A1*	1
MAPK signaling pathway	KEGG:04010	*EGFR*|*FOS*|*KRAS*	3
ErbB signaling pathway	KEGG:04012	*AREG*|*EGFR*|*KRAS*	3
Chemokine signaling pathway	KEGG:04062	*KRAS*	1
p53 signaling pathway	KEGG:04115	*IGF1*	1
mTOR signaling pathway	KEGG:04150	*IGF1*	1
Dorso-ventral axis formation	KEGG:04320	*EGFR*|*KRAS*	2
VEGF signaling pathway	KEGG:04370	*KRAS*	1
Focal adhesion	KEGG:04510	*EGFR*||*IGF1*|	2
Adherens junction	KEGG:04520	*EGFR*	1
Tight junction	KEGG:04530	*KRAS*	1
Gap junction	KEGG:04540	*EGFR*|*KRAS*	2
Toll-like receptor signaling pathway	KEGG:04620	*FOS*	1
Natural killer cell mediated cytotoxicity	KEGG:04650	*KRAS*	1
T cell receptor signaling pathway	KEGG:04660	*FOS*|*KRAS*	2
B cell receptor signaling pathway	KEGG:04662	*FOS*|*KRAS*|	2
Fc epsilon RI signaling pathway	KEGG:04664	*KRAS*	1
Regulation of actin cytoskeleton	KEGG:04810	*EGFR*|*KRAS*	2
Insulin signaling pathway	KEGG:04910	*KRAS*	1
GnRH signaling pathway	KEGG:04912	*EGFR*|*KRAS*	2
Pathways in cancer	KEGG:05200	*EGFR*|*FOS*|*IGF1*|*KRAS*	4
Pancreatic cancer	KEGG:05212	*EGFR*|*KRAS*	2
Endometrial cancer	KEGG:05213	*EGFR*|*KRAS*	2

### 2.5. Association between Endometriosis and Exposure to PCB, Phthalates or BPA

We identified 11 epidemiologic studies related to PCB, phthalate, or BPA exposure and endometriosis. Eight of the studies assessed the relationship between PCB exposure and endometriosis [38,39,40,41,42,43,44], two studies assessed the relationship between phthalate exposure and endometriosis [29,45], one study assessed the relationship between BPA exposure and endometriosis [46], and one study assessed the relationship between phthalate and BPA exposure and endometriosis [47]. Of these studies, eight were case-control studies, one was a cross-sectional study and two were cohort studies. In all of the studies, endometriosis cases were confirmed with a laparoscopic examination and/or biopsy and in nine of the eleven studies controls were also confirmed to be disease free through laparoscopic examination. Controls in the remaining two studies were randomly selected from a list of Group Health Enrollees that were known to not have endometriosis.

All eight of the studies that addressed the relationship between PCB exposure and endometriosis presented individual congener results as well as a measure of total PCBs, the sum of individual congeners. To summarize the main results between PCB exposure and endometriosis, lipid adjusted arithmetic means or geometric means of total PCB exposure were provided for four studies [34,38,41,46], median TEQ values (pg TEQ/g lipid) were provided in two studies [40,43], and median wet weight serum PCB concentrations were calculated in one study [42]. Furthermore, all studies estimated the risk of endometriosis using adjusted logistic regression models with OR and 95% confidence intervals, with the majority of the studies using tertiles or quartiles to compare highest *versus* lowest exposure categories.

Only three of the eight PCB case-control studies found associations between exposure to total PCBs and risk of endometriosis [34,36,41]. Louis *et al.* [36] measured total PCBs (*n* = 62), the sum of estrogenic PCBs (*n* = 12), and the sum of anti-estrogenic PCBs (*n* = 4) in a cohort study of 84 women undergoing laparoscopy (32 endometriosis cases, 52 controls). They found a significant increased risk of endometriosis for the sum of anti-estrogenic PCBs for women in the third tertile (OR = 3.77, 95% CI 1.12–12.68), however, the risk remained elevated but not significant when adjusted for all listed covariates. In a case-control study of 158 women (80 cases and 78 controls), Porpora *et al.* [42] found the GM of total PCBs to be significantly higher in cases than controls (301.3 *vs.* 203.0, *p* < 0.01). The OR of endometriosis risk in the highest tertile of total PCBs compared with the lowest tertile, was 5.63 (95% CI 2.25–14.10). Significant increased risk of endometriosis was also found for PCB congeners 118, 138, 153, and 170. Heiler *et al.* [38] conducted a case-control study of 50 cases (25 with peritoneal endometriosis (PE) and 25 with deep endometriotic (DE) nodules) and 21 controls. Multiple dioxin-like PCBs were measured and expressed as toxic equivalent (TEQ) per gram of serum lipids. Dioxin-like PCB concentrations were higher in women with DE compared to controls {12.4 (10.3 − 14.9) *vs.* 8.5 (6.9 − 10.5), *p* = 0.026} but did not significantly differ for women with PE compared to controls {11.0 (9.1 − 13.3) *vs.* 8.5 (6.9 − 10.5)} and for women with DE compared to women with PE (12.4 *vs.* 11.0).

Four of the PCB case-control studies failed to find significant associations between endometriosis and exposure to individual PCB congeners, total PCBs, or specific sub-groups [38,40,42,43]. Niskar *et al.* [40] conducted a case-control study with 60 confirmed endometriosis cases staged as I (minimal), II (mild), III (moderate), and IV (severe) and 30 controls. Mean lipid-adjusted PCB concentrations were not significantly different (179.98 *vs.* 217.33 *vs.* 194.76 *vs.* 193.37) between stage I–II cases, stage III cases, stage IV cases, and controls, respectively. In the largest case-control study (Trabert *et al.* 2010 [43]), total PCBs (*n* = 20), estrogenic PCBs (*n* = 6), and individual PCB congeners were measured in the serum from 251 cases and 538 controls, matched for age and reference year. Adjusted total and estrogenic PCBs in the highest quartiles were not associated with an increased risk of endometriosis (Total: OR = 1.2, 95% CI 0.6–2.3, Estrogenic: OR = 0.9, 95% CI 0.5–1.4). In two case-control studies measuring median TEQ values (pg TEQ/g lipid) Pauwels *et al.* [43] found no association between endometriosis and the median TEQ values (pg TEQ/g lipid) in cases and controls (29 *vs.* 27) and Tsukino *et al.* [44] found no difference in median TEQ values for endometriosis cases (stage II–IV) and controls (stage 0–I) (cPCBs: 3.40 *vs.* 3.59, PCBs: 4.61 *vs.* 5.14), respectively. The OR of endometriosis risk in the highest quartile of total PCBs compared with the lowest quartile was 0.41 (95% CI 0.14–1.27).

**Figure 4:**
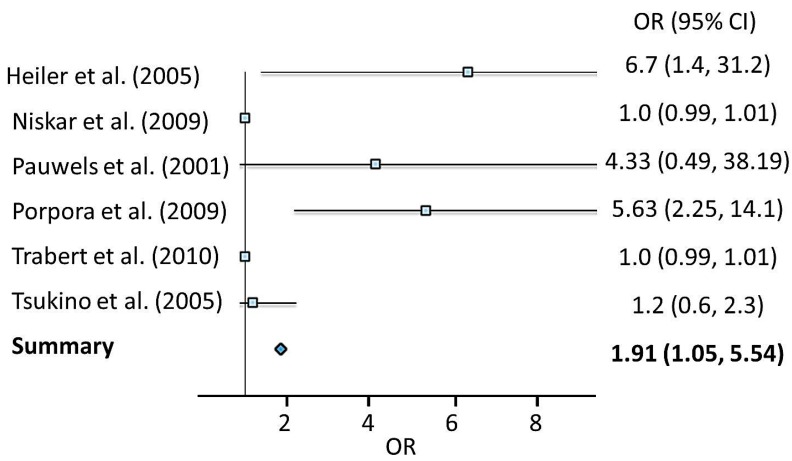
Forest plot of epidemiological studies of the associations between exposure to PCBs and risk of endometriosis.

Like breast cancer, results of the association between PCB exposure and endometriosis in eight epidemiologic studies were inconsistent or conflicting; therefore, we extracted and summarized risk estimates of PCBs on endometriosis from four case control studies using meta-analytic methods. Combining four studies of exposure to PCBs produced a summary risk estimate of 1.91 (95% CI: 1.05–5.54) (Table 4; Figure 4). PCBs exposures were found to be significantly associated with development of endometriosis as a meta-analysis of four studies produced an increased risk of 1.91. However, there is not much confidence in the combined risk estimate of endometriosis with exposure to PCBs because of the lower estimate of CI being barely higher than 1 (1.05).

**Table 4:** Epidemiological Studies of the Association between Exposure to PCBs and Risk of Endometriosis.

Reference, Location	Study Design	Study Population	Measurement of Exposure	Outcomes	Results	Comments	Confounders
Heiler *et al.* [38], Belgium	Case-control study	50 cases: (25 with PE, 25 with DE), 21 controls	Multiple PCBs from serum, 12 dioxin-like PCBs (pg TEQ/g lipids).	Mean serum PCB Range (pg TEQ/g lipids): Controls: 6.9–10.5; PE Cases: 9.1–13.3; DE Cases: 0.3–14.9; Logistic Regression (OR, 95% CI).	Significant risk with DE nodules (OR = 6.7; 95% CI, 1.4–31.2).	Controls did not present for infertility; normal pelvic exam. Cases confirmed with histological exam of lesions.	Adjusted for age, BMI, tobacco consumption, age at menarche, duration of OC use, family history, menstrual cycle regularity, # of children, breast-feeding duration.
Niskar *et al.* [40], USA	Case-control study	60 cases, 30 controls/ 64 controls	Serum total PCBs (ng/g) (*n* = 36).	GM Total PCBs (ng/g lipid): Cases stage I–II (179.98), stage III (217.33), stage IV (194.76), Controls (193.37). Logistic Regression (OR, 95% CI).	No significant differences in GMs (*p* = 0.97). No significant associations (OR = 1.00, 95% CI 0.99–1.01).	Cases confirmed with laparoscopic examination and/or biopsy. 30 controls confirmed with laparoscopy, 27 with infertile partner and 7 with ovulation problems.	Adjusted for age, gravidity, education, income.
Pauwels *et al.* [41], Belgium	Prospective case-control study	42 cases, 27 controls	Multiple PCBs from serum; Total PCBs, TEQ (pg TEQ/g lipid).	Median TEQ (pg TEQ/g lipid): Cases (29), Controls (27). Logistic Regression (OR, 95% CI).	No significant associations found (OR = 4.33, 95% CI 0.49–38.19).	Cases and controls infertile. Endometriosis confirmed with laparoscopic examination.	Age, BMI, alcohol consumption.
Porpora *et al.* [42], Italy	Case-control study	80 cases, 78 controls	Multiple PCBs from serum, Total PCBs.	GM of Total PCBs (ng/g of fat): Cases: 301.3; Controls: 203.0; Logistic Regression (OR, 95% CI).	Total PCB concentrations significantly higher in cases (OR = 5.63, 95% CI 2.25–14.10); Significant increased risk for PCBs 118, 138, 153, and 170 for 2nd and 3rd tertiles when compared to the lowest tertile.	Cases and controls confirmed with laparoscopic examination.	Adjusted for age, BMI, smoking habits, weight modification.
Trabert *et al.* [43], USA	Case-control study	251 cases, 538 controls; matched for age (5 year) and reference year	Multiple PCB congeners in serum (*n* = 20); Total PCBs, Estrogenic PCBs.	Logistic Regression (OR, 95% CI); Quartiles.	No significant associations found.	Cases: Group Health (GH) enrollees with endometriosis diagnosis, Controls: randomly selected from list of GH enrollees.	Adjusted for matching factors, serum lipids, income, alcohol consumption, DDE exposure.
Tsukino *et al.* [44], Japan	Case-control study	139 women: Controls: Stage 0 & I, Cases: Stage II–IV; Stage 0 = 59 Stage I = 22 Stage II = 10 Stage III = 23 Stage IV = 25	Multiple PCBs in serum; Total TEQ values of cPCBs and PCBs.	Median TEQ values (pg TEQ/g lipid); Logistic Regression (OR, 95% CI); Quartiles.	No significant associations found (OR = 1.2, 95% CI 0.6–2.3).	Cases and controls confirmed with laparoscopic examination.	Adjusted for menstrual regularity and average cycle days.

Table 5 lists epidemiological studies of the association between EDCs-phthalate or BPA and endometriosis. No meta-analysis was performed on exposure to BPA or phthalates and endometriosis, because only two studies that met our criteria of selection examined the association between endometriosis and phthalates [39,44]; one study addressed the association between endometriosis and BPA [45], and one study addressed the association between both BPA and phthalates and endometriosis [29]. Besides these two studies, there are several other epidemiological studies that have examined the association between phthalate or BPA exposure and endometriosis [10,29,37,39,46,48,49,50], Table 5. Kim *et al.* [39] measured plasma levels of mono (2-ethylhexyl) phthalate (MEHP) and di-(2-ethylhexyl) phthalate (DEHP) in 97 women with advanced-stage endometriosis and 169 control women. Mean plasma levels of MEHP and DEHP were found to be significantly higher in cases than controls (MEHP: 17.4 *vs.* 12.4, *p* < 0.001, DEHP: 179.7 *vs.* 92.5, *p* = 0.010). In a population-based case-control study conducted by Upson *et al.* [45] 8 urinary phthalate metabolites were measured in 92 surgically-confirmed endometriosis cases and 195 controls. A significant inverse association was found between urinary MEHP and risk of endometriosis (OR = 0.3, 95% CI 0.1–0.7). The ENDO study was designed to assess the relationship between exposure to environmental chemicals and endometriosis. Louis *et al.* [46] analyzed 14 phthalate metabolites and total BPA in urine from 495 women who underwent laparoscopy (operative cohort) and 131 women (population cohort) who underwent pelvic magnetic resonance imaging (MRI) for the assessment of endometriosis. In the operative cohort, GMs of phthalate metabolites were not found to be significantly higher in women with endometriosis, whereas, in the population cohort, GMs of six phthalate metabolites were found to be significantly higher for women with endometriosis and a two-fold or higher increase in ORs was observed for mono-*n*-butyl phthalate (mBP), mono-(2-ethyl-5-carboxyphentyl) phthalate (mECPP), mono-[(2-carboxymethyl) hexyl] phthalate (mCMHP), mono (2-ethyl-5-hydroxyhexyl) phthalate (mEHHP), mono (2-ethyl-5-oxohexyl) phthalate (mEOHP), and mono (2-ethylhexyl) phthalate (mEHP). No significant associations were found for urinary BPA concentrations in either the operative cohort or the population cohort. In a hospital based cross-sectional study, conducted by Itoh *et al.* [51], urinary BPA concentrations were analyzed in 140 women who underwent laparoscopy. The severity of endometriosis was classified into five stages: 0 (*n* = 60), I (*n* = 21), II (*n* = 10), III (*n* = 24), and IV (*n* = 25). Median creatinine adjusted urinary BPA concentrations did not significantly differ by endometriosis stage (0.74 *vs.* 0.93, *p* = 0.24) for stages 0–I and stages II–IV, respectively.

**Table 5:** Epidemiological studies of the association between EDCs-Phthalate or BPA and endometriosis.

EDCs	Biological Samples	Study Population	Outcomes	References
Bisphenol A	Serum	69 fertile women undergoing laparoscopy, Naples, Italy	Detected in cases	Cobellis *et al.* [47
Bisphenol B	Serum	69 fertile women undergoing laparoscopy, Naples, Italy	Detected in cases	Cobellis *et al.* [47]
Phthalate esters	Plasma	220 South Indian women undergoing laparoscopy	Increased risk	Reddy *et al.* [37]
	Serum	108 South Indian women undergoing laparoscopy	Increased risk	Reddy *et al.* [50]
Diethylphthalate	Blood/perit	59 fertile women undergoing laparoscopy	Higher in cases	Cobellis *et al.* [47]
Monoethylphthalate	Blood/peri-toneal fluid	59 fertile women undergoing laparoscopy	No association	Cobellis *et al.* [47]
Monobutylphthalate	Urine	1227 women from the NHANES study, United States	No association	Calafat *et al.* [10]
	Urine	109 women undergoing laparotomy, Taiwan	Increased in cases	Huang *et al.* [48]
Monobutylphthalate	Urine	1227 women from the NHANES study, USA	No association	Calafat *et al.* [10]
	Urine	109 women undergoing laparotomy, Taiwan	Increased in cases	Huang *et al.* [48]

**Table 6:** Genes interacting with polychlorinated biphenyls in endometriosis.

IUPAC Name (Congener Number)	Interacting Genes
Polychlorinated Biphenyls	19 genes: *AKR1C3* | *ANKRD1* | *AREG* | *ARNT* | *CYP19A1* | *DUSP1* | *ESR2* | *FBN1* | *FOS* | *GREB1* | *IGFBP1* | *KRAS* | *NR2C2* | *NR3C1* | *PAPPA* | *PTGER4* | *STC2* | *TGFB2* | *THRA*
2,4,4ʹ-Trichlorobiphenyl (28)	2 genes: *ESR2* | *NR3C1*
3,4,3ʹ,4ʹ-Tetrachlorobiphenyl (77)	11 genes: *ARNT* | *DDX5* | *ESR2* | *FKBP5* | *ITGB8* | *KLF13* | *MAOB* | *NR1D2* | *PRLR* | *SULF2* | *TXNIP*
2ʹ,3,3ʹ,4ʹ,5-Pentachloro-4-hydroxybiphenyl (4ʹ-OH-PCB-86; 4-hydroxy-2,2ʹ,3ʹ,4ʹ,5ʹ-pentachlorobiphenyl )	25 genes: *ABCC9* | *BRD8* | *CD55* | *CNR1* | *ELAVL1* | *ERRFI1* | *FKBP5* | *IFNGR1* | *IGFBP1* | *MED1* | *MED4* | *MTA1* | *NCOA6* | *NR2C1* | *NR3C1* | *NR3C2* | *NR4A1* | *NRP1* | *PRLR* | *SLC16A6* | *SPARCL1* | *SST* | *TAGLN* | *THRA* | *TNC*
2,2ʹ,4,6,6ʹ-Pentachlorobiphenyl (104)	2 genes: *EGFR* | *FOS*
3,4,5,3ʹ,4ʹ-Pentachlorobiphenyl (126)	36 genes: *AREG* | *CD55* | *CXCL14* | *CYP19A1* | *DUSP1* | *ENPP1* | *FBN1* | *GPX3* | *HBEGF* | *HSD17B1* | *HSD17B2* | *IGF1* | *IGFBP1* | *IGFBP6* | *IL1R1* | *IMPA2* | *MAOA* | *MAOB* | *MED1* | *NEDD4L* | *NR3C1* | *OSR2* | *PRLR* | *RASL11A* | *SEPP1* | *SLC20A1* | *SLC40A1* | *SLC7A8* | *SPARCL1* | *SRD5A1* | *SRD5A2* | *SST* | *STC2* | *TAGLN* | *TGFB2* | *TXNIP*
2,2ʹ,3ʹ,4,4ʹ,5-Hexachlorobiphenyl (137)	10 genes: *FBLN1* | *FOS* | *HBEGF* | *IGF1* | *NEFM* | *PRL* | *SLC16A6* | *SRD5A1* | *SRD5A2* | *SST*
2,3,6,2ʹ,3ʹ,6ʹ-Hexachlorobiphenyl (136)	2 genes: *AR* | *CYP2B1*
2,4,5,2ʹ,4ʹ,5ʹ-Hexachlorobiphenyl (153)	18 genes: *CYP19A1* | *DCSTAMP* | *EGFR* | *ESR2* | *FOS* | *HSD17B1* | *HSD17B2* | *IFIT1* | *IGF1* | *ITGB8* | *MAOB* | *NR3C1* | *SEPP1* | *SLC16A6* | *SRD5A1* | *SRD5A2* | *SST* | *TXNIP*
17β Estradiol	114 genes: *ABCC9* | *ABI3BP* | *ACTA2* | *AKR1C1* | *AKR1C2* | *AKR1C3* | *ANKH* | *ANKRD1* | *AREG* | *ARHGAP28* | *ARNT* | *BMP7* | *C10ORF10* | *C1R* | *CCNE2* | *CD55* | *CFD* | *CLDN1* | *CNIH3* | *CNR1* | *CPM* | *CXCL14* | *CYB5A* | *CYP19A1* | *CYP26A1* | *DDX5* | *DICER1* | *DIO2* | *DKK1* | *DUSP1* | *EGFR* | *ELAVL1* | *ERRFI1* | *ESR2* | *FBLN1* | *FBN1* | *FKBP5* | *FOS* | *GPX3* | *GREB1* | *HDAC1* | *HDAC2* | *HERC5* | *HS3ST3B1* | *HSD17B1* | *HSD17B2* | *IDO1* | *IFIT1* | *IGF1* | *IGFBP1* | *IGFBP6* | *IHH* | *IL15* | *IL1R1* | *IL7R* | *ITGA2* | *ITGB1* | *ITGB8* | *KLF13* | *KLF9* | *KRAS* | *LMOD1* | *LTF* | *MAOA* | *MAOB* | *MED1* | *MED14* | *METTL7A* | *MIR21* | *MYLIP* | *NCOA1* | *NCOA6* | *NCOR1* | *NEDD4L* | *NR2F2* | *NR3C1* | *NR3C2* | *NR4A1* | *NR5A1* | *NRP1* | *NTRK3* | *OLFM4* | *OSR2* | *PAPPA* | *PGR* | *PRL* | *PRLR* | *PTGER2* | *PTGER4* | *RARB* | *RASGRP1* | *RGS4* | *RORB* | *RXFP1* | *SEPP1* | *SLC16A6* | *SLC1A1* | *SLC20A1* | *SLC40A1* | *SLC7A8* | *SMPDL3A* | *SPARCL1* | *SRD5A2* | *STC2* | *SULF2* | *TACSTD2* | *TAGLN* | *TGFB2* | *THRA* | *TNC* | *TOB1* | *TRH* | *TXNIP* | *VCAN ZEB2*
Dibutyl phthalate	71 genes: *ABI3BP* | *ACTA2* | *AKR1C1* | *ANKRD1* | *AREG* | *ARNT* | *BMP7* | *BRD8* | *C1R* | *CCNE2* | *CD55* | *CLDN1*| *CNR1* | *COPS2* | *CYB5A* | *CYP19A1* | *CYP26A1* | *DDX5* | *DICER1* | *DUSP1* | *EGFR* | *ELAVL1* | *ENPP1* | *ERRFI1* | *ESR2* |*FKBP5* | *FOS* | *GPX3* | *HDAC1* | *HSD17B1* | *IGF1* | *IL1R1* | *ITGB1* | *ITGB8* | *KLF9* | *LMOD1* | *MAOA* | *MAOB* | *MED1* |*MED14* | *MED17* | *NR1D2* | *NR2F2* | *NR2F6* | *NR3C1* | *NR4A1* | *NR5A1* | *NRP1* | *OSR2* | *PAPPA* | *PGR* | *PRLR* | *PTGER2* |*PTGER4* | *RASL11A* | *SEPP1* | *SLC16A6* | *SLC20A1* | *SLC40A1* | *SLC7A8* | *SMPDL3A* | *SRD5A1* | *STC2* | *SUCLG2* |*SULF2* | *TAGLN* | *TGFB2* | *THRA* | *TOB1* | *TXNIP* | *VCAN.*
Diethylhexyl phthalate	29 genes: *CNR1* | *CYP19A1* | *CYP26A1* | *EGFR* | *ESR2* | *FKBP5* | *FOS* | *HERC5* | *IGF1* | *IGFBP1* | *ITGB1* | *MAOA* | *NCOA1* | *NR3C1* | *NR4A1* | *NR5A1* | *PAX2* | *PRL* | *PRLR* | *PTGER2* | *PTGER4* | *SRD5A1* | *TGFB2* | *DUSP1* | *FMO2* | *GPX3* | *MED1* | *NCOR1* | *TXNIP*
Dibutyl phthalate and diethyl-hexyl phthalate	22 genes: *CNR1* | *CYP19A1* | *CYP26A1* | *DUSP1* | *EGFR* | *ESR2* | *FKBP5* | *FOS* | *GPX3* | *IGF1* | *ITGB1* | *MAOA* | *MED1* | *NR3C1* | *NR4A1* | *NR5A1* | *PRLR* | *PTGER2* | *PTGER4* | *SRD5A1* | *TGFB2* | *TXNIP*
Bisphenol A	80 genes: *ABCC9* | *ACTA2* | *AREG* | *ARHGAP28* | *ARNT* | *BMP7* | *BRD8* | *CCNE2* | *COPS2* | *CYB5A* | *CYP19A1* | *CYP26A1* | *DDX5* | *DICER1* | *DIO2* | *DUSP1* | *EGFR* | *ELAVL1* | *ENPP1* | *ERRFI1* | *ESR2* | *FKBP5* | *FOS* | *GPX3* | *GREB1* | *HDAC1* | *HDAC2* | *HSD17B1* | *HSD17B2* | *IFNGR1* | *IGF1* | *IGFBP1* | *IGFBP6* | *ITGB8* | *KLF9* | *KRAS* | *LMOD1* | *LTF* | *MAOA* | *MED1* | *MED14* | *MED16* | *MED17* | *MED4* | *MIR21* | *NCOA1* | *NCOR1* | *NR2C1* | *NR2F2* | *NR3C1* | *NR3C2* | *NR4A1* | *NR5A1* | *NRP1* | *OLFM4* | *PAPPA* | *PGR* | *PRL* | *PRLR* | *PTGER2* | *PTGER4* | *RASGRP1* | *RASL11A* | *RGS4* | *RORB* | *SLC1A1* | *SLC40A1* | *SLC7A8* | *SMPDL3A* | *SRD5A1* | *SRD5A2* | *SST* | *STC2* | *SULF2* | *TACSTD2* | *TAGLN* | *TGFB2* | *THRA* | *TNC* | *VCAN*

**Figure 5:**
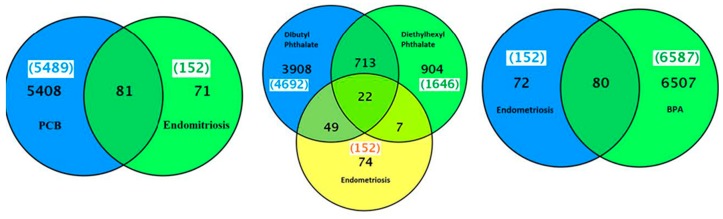
A Venn diagram of list of genes common between endometriosis and PCBs, phthalates or bisphenol A.

Several hundred genes were altered by exposure to PCBs, phthalate or BPA (Figure 5). The genes related to PCB and PCB congeners-3,4,3′,4′-tetrachlorobiphenyl (77), 3,4,5,3′,4′-pentachlorobiphenyl (126), and 2,4,5,2′,4′,5′-hexachlorobiphenyl (153) in endometriosis were 19, 11, 36 and 18, respectively (Table 6). All these PCBs or their congener-related endometriosis genes were also associated with 17β-estradiol. The top interacting genes with PCBs and endometriosis were *ESR2*, *NR3C1*, *CYP19A1*, *EGFR*, *FKBP5*, *ITGB8*, *MAOB*, *PGR*, *PRLR*, *SLC16A6*, *SST*, and *TXNIP*. There were 80 common genes found between BPA and endometriosis (Figure 5). The two phthalates with the most gene interactions were: dibutyl phthalate and diethylhexyl phthalate. The genes related to dibutyl phthalate and diethylhexyl phthalate were 4692 and 1646, respectively. There were 71 common genes associated between dibutyl phthalate and endometriosis and 29 common genes between diethylhexyl phthalate and endometriosis (Figure 5). There were 22 genes in common between both phthalates-dibutyl phthalate and diethylhexyl phthalate, and endometriosis, as shown in Table 6. Interactions among these genes are shown in Figure S2. Enrichment pathway analysis revealed that some of these genes are part of: (1) pathways in cancer (KEGG:05200); (2) signal transduction (REACT:111102); and (3) MAPK signaling pathway ( KEGG:04150) (Table 3).

### 2.6. Integration of Genes Overlapped among EDCs, Breast Cancer and Endometriosis

Integration of genes associated with exposure to PCBs, and breast cancer and endometriosis based enriched disease analysis showed that there were 16 endometriosis genes overlapped with breast neoplasms—*AREG*, *C10ORF10*, *CLDN1*, *CYP19A1*, *DKK1*, *EGFR*, *ESR2*, *FOS*, *IGF1*, *KRAS*, *NCOA1*, *NCOR1*, *NR2F6*, *PGR*, *RARB*, and *STC2* (Table 2 and Table 6). All of these genes were also associated with estrogen in breast neoplasms. Out of these 16 genes, there were 14 genes—*AREG*, *CLDN*, *CYP19A1*, *DKK1*, *EGFR*, *ESR2*, *FOS*, *IGF1*, *KRAS*, *NCOA1*, *NCOR1*, *NR2F6*, *PGR*, *RARB*, and *STC2*—common among 17β-estradiol, breast cancer, and endometriosis (Table 2, Table 6 and Table 7). Total PCBs associated with *AREG*, *CYP19A1*, *ESR2*, *FOS*, *KRAS* and *STC2* genes; PCB 126 associated with *AREG*, *CYP19A1*, and *STC2* genes and PCB 15 associated with *CYP19A1*, *EGFR*, *ESR2*, *FOS*, and *IGF1* genes overlapped with 17β-estradiol, breast cancer, and endometriosis (Table 2, Table 6 and Table 7). Similarly, we identified dibutyl phthalate and diethyl-hexyl phthalate associated overlapping genes with 17β-estradiol, breast cancer, and endometriosis: *AREG*, *CLDN1*, *CYP19A1*, *EGFR*, *ESR2*, *FOS*, *IGF1*, *NR2F6*, *PGR and STC2*; and *CYP19A1*, *EGFR*, *ESR2*, *FOS*, *IGF1*, and *NCOA1*. There were five common overlapped genes between these two phthalates, 17β-estradiol, breast cancer and endometriosis: *CYP19A1*, *EGFR*, *ESR2*, *FOS*, and *IGF1*. We also identified another 11 EDC–BPA associated genes that were common among 17β-estradiol, breast cancer and endometriosis: *AREG*, *CYP19A1*, *EGFR*, *ESR2*, *FOS*, *IGF1*, *KRAS*, *NCOA1*, *NCOR1*, *PGR*, and STC2. Five genes—CYP19A1, EGFR, ESR2, FOS, and IGF1—were common among all three EDCs–PCBs, phthalates and BPA, 17β-estradiol, breast cancer, and endometriosis. For the gene ontology terms associated with each gene, please see Table 8.

Since both of these diseases are dependent on unopposed estrogen for their growth, we examined whether estrogen receptor signaling pathway genes are common among estrogen, EDCs, breast cancer and endometriosis. PCBs and congeners 3,4,5,3′,4′-pentachlorobiphenyl (126) and 2,4,5,2′,4′,5′-hexachlorobiphenyl (153) were associated with some of the same estrogen receptor signaling pathway genes—*AR*, *ESR1*, *ESR2*, *NCOA3*, and *PPARGC1B*; *AR*, *BRCA1*, *ESR1*, *IGF1*, and *PAK1*; and *AR*, *BRCA1*, *CTNNB1*, *ESR1*, *ESR2*, *IGF1*, and *SRC*, respectively (Table 7). The following were also observed with 17β-estradiol—*AR*, *BRCA1*, *CCNE1*, *CTNNB1*, *ESR1*, *ESR2*, *FHL2*, *FOXA1*, *IGF1*, *NCOA1*, *NCOA2*, *NCOA3*, *NRIP1*, *PAK1*, *PGR*, *PHB*, *PPARGC1B*, *RB1*, *SFRP1*, *SRC*, and *ZNF366*. Similarly, common genes of estrogen receptor signaling pathways were also observed with another three EDCs. Dibutyl phthalate associated genes, *AR*, *BRCA1*, *CCNE1*, *CTNNB1*, *ESR1*, *ESR2*, *FHL2*, *HEYL*, *IGF1*, *PGR*, *RB1*, and *SRC*; and diethylhexyl phthalate associated genes, *AR*, *CTNNB1*, *ESR1*, *ESR2*, *IGF1*, *NCOA1*, and *PPARGC1B*, and BPA associated *AR*, *BRCA1*, *CCNE1*, *CTNNB1*, *ESR1*, *ESR2*, *FHL2*, *IGF1*, *NCOA1*, *NCOA2*, *NCOA3*, *NRIP1*, *PAK1*, *PGR*, *PHB*, *RB1*, *SFRP1*, *SIRT1*, and *SRC*, are also associated with 17β-estradiol in breast neoplasms (Table 7).

**Table 7:** EDCs observed in breast neoplasms that are associated with estrogen responsive gene interactions, endometriosis, and inflammation.

EDC Interacting with Genes in Breast Neoplasms	Steroid Hormone Receptor Signaling Pathway	Endometriosis	Inflammation
17β Estradiol	*AR*, *BRCA1*, *CCNE1*, *CTNNB1*, *ESR1*, *ESR2*, *FHL2*, *FOXA1*, *IGF1*, *NCOA1*, *NCOA2*, *NCOA3*, *NRIP1*, *PAK1*, *PGR*, *PHB*, *PPARGC1B*, *RB1*, *SFRP1*, *SRC*, *ZNF366*	*AREG*, *CLDN1*, *CYP19A1*, *DKK1*, *EGFR*, *ESR2*, *FOS*, *IGF1*, *KRAS*, *NCOA1*, *NCOR1*, *PGR*, *RARB*, *STC2*	*AHR*, *CSF3*, *CXCL2*, *CXCL8*, *HMOX1*, *IFNG*, *IL1B*, *IL6*, *LEP*, *MIF*, *MMP9*, *NOS2*, *NOS3*, *PARP1*, *PTGS2*, *SOD2*, *TFRC*, *TNF*
PCBs	*AR*, *ESR1*, *ESR2*, *NCOA3*, *PPARGC1B*	*AREG*, *CYP19A1*, *SR2*, *FOS*, *KRAS*, *STC2*	*AHR*, *CXCL2*, *HMOX1*, *IFNG*, *IL6*, *PTGS2*, *SOD2*, *TNF*
3,4,5,3′,4′-Pentachlorobiphenyl (126)	*AR*, *BRCA1*, *ESR1*, *IGF1*, *PAK1*	*AREG*, *CYP19A1*, *STC2*	*AHR*, *CXCL8*, *HMOX1*, *IL1B*, *IL6*, *MMP9*, *NOS2*, *NOS3*, *PARP1*, *PTGS2*, *TNF*
2,4,5,2′,4′,5′-Hexachlorobiphenyl (153)	*AR*, *BRCA1*, *CTNNB1*, *ESR1*, *ESR2*, *IGF1*, *SRC*	*CYP19A1*, *EGF*, *ESR2*, *FOS*, *IGF1*	*AHR*, *IFNG*, *IL1B*, *PARP1*, *PTGS2*, *TNF*
Dibutyl Phthalate	*AR*, *BRCA1*, *CCNE1*, *CTNNB1*, *ESR1*, *ESR2*, *FHL2*, *HEYL*, *IGF1*, *PGR*, *RB1*, *SRC*	*AREG*, *CLDN1*, *CYP19A1*, *EGFR*, *ESR2*, *FOS*, *IGF1*, *R2F6*, *PGR*, *STC2*	*AHR*, *CXCL8*, *HMOX1*, *IL1B*, *IL6*, *MIF*, *MMP9*, *PARP1*, *SOD2*, *TFRC*, *TNF*
Diethylhexyl Phthalate	*AR*, *CTNNB1*, *ESR1*, *ESR2*, *IGF1*, *NCOA1*, *PPARGC1B*	*CYP19A1*, *EGFR*, *ESR2*, *FOS*, *IGF1*, *NCOA1*	*AHR*, *CSF2*, *CXCL8*, *IFNG*, *LEP*, *MMP9*, *SOD2*, *TNF*
Bisphenol A	*AR*, *BRCA1*, *CCNE1*, *CTNNB1*, *ESR1*, *ESR2*, *FHL2*, *IGF1*, *NCOA1*, *NCOA2*, *NCOA3*, *NRIP1*, *PAK1*, *PGR*, *PHB*, *RB1*, *SFRP1*, *SIRT1*, *SRC*	*AREG*, *CYP19A1*, *EGFR*, *ESR2*, *FOS*, *IGF1*, *KRAS*, *NCOA1*, *NCOR1*, *PGR*, *STC2*	*AHR*, *CSF2*, *HMOX1*, *IFNG*, *IL1B*, *IL6*, *LEP*, *MIF*, *MMP9*, *NOS2*, *NOS3*, *PARP1*, *PTGS2*, *SOD2*, *TNF*

**Table 8:** Integration of changes in the expression of genes showing common genes modified in EDCs, breast cancer and endometriosis. The underlined gene names show a total of five genes that were common among all three EDCs (PCBs, phthalate and bisphenol A), breast cancer, and endometriosis. Environmentally responsive genes are indicated in database column.

Gene Name	Gene ID	Location	Database *	Gene Function
*AREG*	374	4q13–q21	E	Amphiregulin
*CYP19A1*	1588	15q21.1	E	Cytochrome P450, family 19, subfamily A, polypeptide 1
*EGFR*	1956	7p12	E	Epidermal growth factor receptor
*ESR2*	3468	14q23.2	H	Estrogen receptor 2 (ER β)
*FOS*	2353	14q24.3	E	v-Fos FBJ murine osteosarcoma viral oncogene homolog
*IGF1*	3479	12q22-q23	E	Insulin-like growth factor 1 (somatomedin C)
*KRAS*	6407	12p12.1	H	Kirsten rat sarcoma viral oncogene homolog
*NCOA1*	7668	2p23	H	Nuclear receptor coactivator 1
*NCOR1*	7672	17p11.2	H	Nuclear receptor corepressor 1
*PGR*	5241	11q22-q23	E	Progesterone receptor
*STC2*	11374	5q35.1	H	Stanniocalcin 2

* (E): Environmental responsive gene based on Environmental Genome Project; (H): HGNC database.

Another factor that appears to be common in both diseases is inflammation. Therefore, we also examined whether inflammation associated genes are common among estrogen, EDCs, and breast cancer. PCBs and congeners 3,4,5,3′,4′-pentachlorobiphenyl (126) and 2,4,5,2′,4′,5′-hexachlorobiphenyl (153) were associated with the following inflammation related genes—*AHR*, *CXCL2*, *HMOX1*, *IFNG*, *IL6*, *PTGS2*, *SOD2*, and *TNF*; *AHR*, *CXCL8*, *HMOX1*, *IL1B*, *IL6*, *MMP9*, *NOS2*, *NOS3*, *PARP1*, *PTGS2*, and *TNF*; and *AHR*, *IFNG*, *IL1B*, *PARP1*, *PTGS2*, and *TNF*, respectively (Table 7). Dibutyl phthalate, diethyl-hexyl phthalate and BPA-associated set of inflammation-related genes were *AHR*, *CXCL8*, *HMOX1*, *IL1B*, *IL6*, *MIF*, *MMP9*, *PARP1*, *SOD2*, *TFRC*, and *TNF*; *AHR*, *CSF2*, *CXCL8*, *IFNG*, *LEP*, *MMP9*, *SOD2*, and *TNF*; and *AHR*, *CSF2*, *HMOX1*, *IFNG*, *IL1B*, *IL6*, *LEP*, *MIF*, *MMP9*, *NOS2*, *NOS3*, *PARP1*, *PTGS2*, *SOD2*, and *TNF*, respectively. All of these genes were also associated with 17β-estradiol in breast neoplasms. In summary, EDC associated set of genes from inflammation pathways in breast neoplasms are estrogen responsive.

### 2.7. Literature Based Validation of Genes Showing Links between Endometriosis and Breast Cancer

The set of estrogen responsive genes from EDCs, environmental, inflammation, and toxicogenomics showing a link between endometriosis and breast cancer is shown in Table 7. Research supporting the potential involvement and importance of all EDC responsive common genes in breast cancer and endometriotic lesions was found in the literature and human genome databases. The search of the environmental genome project databases showed that six genes out of 12 PCBs associated genes—*AREG*, *CYP19A1*, *EGFR*, *FOS*, *IGF1*, and *PGR* were environmentally responsive genes (Table 8). These common genes were then compared to a curated list of genes in PCB exposed human cell lines. PCB congeners 77 and 153 increased the expression of the following estrogen responsive genes *AREG*, *CYP19A1*, *EGFR*, *ESR2*, *FOS*, *IGF1*, *KRAS*, *NCOA1*, *NCOR1*, *NR2F6*, *PGR*, *STC2* [52]. The expression of estrogen responsive genes common to breast cancer: *AREG*, *CYP19A1*, *EGFR*, *ESR2*, *FOS*, *IGF1*, *KRAS*, *NCOA1*, *NCOR1*, *NR2F6*, *PGR*, *STC2* genes was upregulated in human endometriosis lesions [53,54,55].

**Figure 6:**
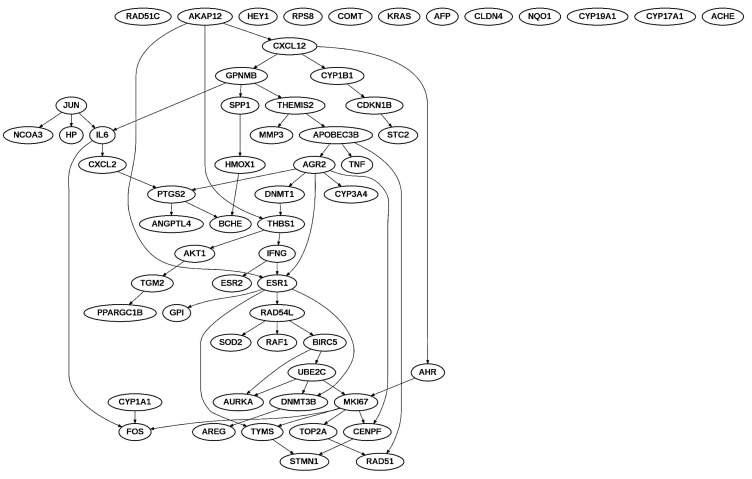
Interaction of common genes between estrogen, PCBs and breast neoplasms—*AREG*, *CYP19A1*, *EGFR*, *ESR2*, *FOS*, *IGF1*, *KRAS*, *NCOA1*, *NCOR1*, NR2F6, *PGR*, and *STC2*.

**Figure 7:**
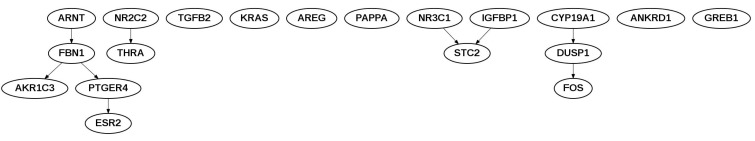
Identification of the maximum likelihood structure of PCBs associated genes in breast neoplasm using the Bayesian network analysis on the Cancer Genome Atlas (TCGA) Research Network data.

We also analyzed the interaction among *AREG*, *CYP19A1*, *EGFR*, *ESR2*, *FOS*, *IGF1*, *KRAS*, *NCOA1*, *NCOR1*, *NR2F6*, *PGR*, and *STC2* genes using enrichment pathway analysis (Figure 6). In order to investigate connections between PCBs responsive gene lists in breast cancer and endometriosis, we performed Bayesian network analysis. The Bayesian network analysis on the Cancer Genome Atlas (TCGA) Research Network data available through cbioportal.org identified the maximum likelihood structure of PCBs associated genes in breast neoplasms (Figure 7).

Figure 7 shows plausible interactions among genes. Parents of a variable in Bayesian networks are defined as variables that arcs are originated to that variable. For example, in Figure 7, parents of the gene *BCHE* are *PTGS2* and *HMOX1*. Ancestors of a variable are all the parents of the variable, all parents of parents, and so on. Arcs in Figure 7 indicate correlations and they indicate Markov conditions. In Figure 7, from the arcs, the relationship between *PTGS2* and *BCHE* was the strongest among all pairwise relationships, but also they formed a special Y structure [56] that indicates plausible causality, *i.e.*, *PTGS2* regulating *BCHE*. Similarly we have analyzed mRNA expression endometriosis data (Figure 7). These genes were more sparsely connected.

Some of the common estrogen responsive interacting genes are part of steroid hormone biosynthesis; metabolic pathways; MAPK signaling pathway; ErbB signaling pathway; chemokine signaling pathway; p53 signaling pathway; mTOR signaling pathway; VEGF signaling pathway; focal adhesion; adherens junction; tight junction; gap junction; toll-like receptor signaling pathway; natural killer cell mediated cytotoxicity; T cell receptor signaling pathway; B cell receptor signaling pathway; Fc epsilon RI signaling pathway; regulation of actin cytoskeleton; insulin signaling pathway; GnRH signaling pathway; and pathways in cancer (Table 3). We also compared these common genes to a curated list of genes in breast cancer, endometriosis as well as EDC exposed populations. The search of the environmental databases showed that some of these common genes were environmentally responsive. All these EDC associated set of genes are estrogen responsive (Table 8). All these PCB, Phthalate and BPA associated common genes are altered in human breast tumor, uterine tumor tissues and endometriosis lesions (Table 8).

## 3. Discussion

In the present study, we focused on developing an integrative approach to elucidate the role of EDCs (PCBs, phthalates and BPA) that contributed to the risk of breast cancer and endometriosis using environmental epidemiologic evidence and molecular signatures. Women with endometriosis have been implicated to develop certain types of cancer, including breast and ovarian cancer [57]. Although several molecular and environmental risk factors are common to endometriosis and breast cancer, the results of epidemiologic studies have been inconsistent on directly linking endometriosis with breast cancer. Both of these diseases are dependent on unopposed estrogen for their growth. Endometrial tissue shows elevated activity of aromatase, and this enzyme is a key for the biosynthesis of estrogens [58]. Our meta-analysis showed that exposure to estrogen mimicking EDCs-PCBs increased summary risk of both breast cancer and endometriosis. Using our bioinformatics method, we further evaluated the relationship between endometriosis and breast cancer, and EDCs. Our bioinformatics approach was able to identify genes with the potential to be involved in interaction with PCBs and other EDCs–phthalates and BPA that may be important to the development of breast cancer and endometriosis. We identified six PCBs associated genes—*AREG*, *CYP19A1*, *EGFR*, *FOS*, *IGF1*, and *PGR*—that are environmentally responsive. Similarly, we also observed dibutyl phthalate and diethyl-hexyl phthalate associated with five common genes—*CYP19A1*, *EGFR*, *ESR2*, *FOS*, and *IGF1*—in breast cancer and endometriosis; and BPA associated 11 genes—*AREG*, *CYP19A1*, *EGFR*, *ESR2*, *FOS*, *IGF1*, *KRAS*, *NCOA1*, *NCOR1*, *PGR*, and STC2—that were common in both breast cancer and endometriosis. Five genes—*CYP19A1*, *EGFR*, *ESR2*, *FOS*, and *IGF1*—were common among all three EDCs–PCB 153, phthalates and BPA, breast cancer, and endometriosis. All five common genes are modified in human breast tumor, uterine tumor tissues, and endometriosis lesions. All of these genes are estrogen responsive. These findings suggest that the increased risk associated with endometriosis may be due to common environmental and molecular risk factors between endometriosis and breast cancer.

Experimental animal and human studies have indicated that EDCs have the ability to cause endocrine toxicity. For example, exposure to PCBs has been reported to show a significant delay in puberty in boys. De-feminization, early secondary breast development, or menarche have been reported in girls exposed to phthalates [4,5,6,7,59]. Despite existing debates over the form and amount of BPA to which developing and adult humans are exposed, there is considerable data indicating that exposure of humans to BPA is associated with increased risk for breast cancer and reproductive dysfunctions [3,4]. Postmenopausal women with high serum levels of BPA and mono-ethyl phthalate have been reported to elevate breast density, one of the risk factors for breast cancer [36]. These findings are consistent with parallel research in experimental models [19,20,21,22]. For example, fetal bisphenol A exposure induces the development of preneoplastic and neoplastic lesions in the mammary gland in rats [22]. Fetal exposure of BPA significantly increases susceptibility to DMBA to produce mammary tumors in mice [21]. BPA has also been reported to promote tumor growth of human breast cancer cells-MCF-7 in ovariectomized NCR nu/nu female mice. Women with the lack of detoxifying enzymes are at higher risk for breast cancer due to excess exposures to polychlorinated dioxins and certain PCBs. who. A landmark UN report assessing effects of human exposure to hormone-disrupting chemicals acknowledges that approximately 800 chemicals are suspected to act as endocrine disruptors or mimic natural hormones or disrupt hormone regulation [6]. This report highlights that there are some associations between exposure to many of the endocrine disruptors, particularly, estrogen-mimicking chemicals and an increased risk of breast cancer in women. Exposure to EDCs, such as, PCBs and BPA during early development of the breast, endometrium, and prostate can alter their development, and possibly contribute to the susceptibility to diseases through effects on stem cells.

Breast cancer and endometriosis are complex chronic diseases and they are not caused by one agent or one environmental factor. The majorities of the epidemiologic studies have largely focused on a single EDC and have ignored the possibility that multiple environmental agents may act in concert. It is important to consider that during the development of an individual from the single cell to prenatal stages to adolescent to adulthood and through the complete life span, humans are exposed to countless environmental EDCs. Like genes, environmental factors also interact among themselves. A single exposure to an EDC alone cannot explain the development of a complex chronic disease, like breast cancer, rather it appears that exposure to multiple EDCs across the lifespan and their interactions influence the development of breast cancer in an individual. A recent study from Spain lends support to the above concept. They have shown that the body burden of lipophilic estrogenic organohalogen chemicals through cumulative exposure is associated with breast cancer risks [60]. The temporal and spatial environmental modulations of the normal genetic and phenotypic changes in a cell lead to the development of a particular type of disease phenotype. However, the majority of epidemiologic studies measured EDC exposures later in a woman’s life, when the breast or endometrium tissue is less vulnerable. In-utero exposure to the estrogenic anti-miscarriage compound-diethylstilboestrol (DES) underlines the importance of early life EDC exposure in breast cancer development and is apparent from the recent report showing elevated breast cancer risks in the daughters of exposed women [61]. Given the proven contribution of unopposed estrogens in the development of breast cancer and endometriosis, it is biologically plausible that less potent EDCs may also contribute to risks of chronic diseases, such as breast cancer and endometriosis [59].

To date, most research on the endometriosis connection to breast cancer development has investigated only a handful of mechanisms and pathways. Genes involved in estrogen biosynthesis, metabolism, estrogen signaling pathway and signal transduction have been suggested to affect susceptibility of breast cancer and endometriosis. In our study we found that five common estrogen responsive genes, including CYP19A1 and ESR2 that were associated with all three EDCs-PCBs, phthalates and BPA, breast cancer, and endometriosis. ESR is an important molecular risk factor in the pathogenesis of breast cancer [62]. We examined the association of estrogen receptor ESR2 and estrogen biosynthesis enzyme, aromatase, CYP19A1 with endometriosis and breast cancer. Both mRNA and protein levels of estrogen receptor 2 (ESR2) were found higher in endometriotic tissue [63]. Increased expression of aromatase has been found in breast tumors [64]. In women with endometriosis, elevated tissue levels of 17β-estradiol due to increased aromatase activity are found [65]. We also observed association of *EGFR*, *FOS* and *IGF1* genes with EDCs, endometriosis and breast cancer. Increased circulating IGF1 level is associated with an increased risk of breast cancer [66]. Another common gene identified in both endometriosis and breast cancer in this study was stanniocalcin 2 (*STC2*). This is a downstream target of estrogen signaling pathways [67]. The expression of *STC2* is induced in MCF-7 cells and the endometrial gland of women by 17β-estradiol and in breast tumors [68,69]. Modified expression of these genes is known to be involved in breast cancer pathways and include mTOR signaling pathway, focal adhesion, VEGF signaling pathway, and ErbB signaling pathway. However, the link of these common genes between these two diseases and EDCs does not prove that one causes the other. Furthermore, our study also revealed that PCBs and congeners 3,4,5,3′,4′-pentachlorobiphenyl (126) and 2,4,5,2′,4′,5′-hexachlorobiphenyl (153) are associated with some of the same estrogen receptor signaling pathway genes in breast neoplasm that are also observed with 17β-estradiol. Similarly, common genes of estrogen receptor signaling pathways were also observed with EDCs–dibutyl phthalate; diethylhexyl phthalate; and BPA and breast neoplasms that are also observed with 17β-estradiol. These finding support genes identified in this study that are highly likely to be involved in estrogen biosynthesis and estrogen signaling pathway to contribute to the susceptibility of breast cancer and endometriosis.

Inflammation is another factor that appears to be common in both breast cancer and endometriosis. Findings of this study showed that EDCs associated with genes involved in inflammation pathways were also associated with 17β-estradiol in breast neoplasms. The role of estrogen in inflammation is complex. On one hand, studies reported suppression of inflammation with increased estrogen in animal models of chronic inflammatory diseases. On the other hand, estrogen has been shown to have proinflammatory effects in some human chronic autoimmune diseases. Estrogen induces proinflammatory cytokines, such as interleukin-1β (IL-1β) and tumor necrosis factor α (TNF-α), and a number of other inflammation associated genes [60], which were also associated with EDCs as observed in this study. Inflammation-mediated oxidative stress is involved in the development of both of these diseases [60]. Prostaglandin E2 is upregulated in endometriosis as a result of inflammation, which increases estrogen synthesis by up regulating aromatase. Therefore a proinflammatory milieu can also directly increase estrogen production and inflammation may work in conjunction with or in addition to EDCs exposure in the development of breast cancer in women with endometriosis [70].

There are several strengths of the meta-analysis of EDCs associated with breast cancer or endometriosis. The use of the general variance based method gave more weight to larger studies, considered confounding, and limited the number of studies excluded because of missing data. Most studies used interview data to assess exposure, providing a more direct accounting of exposure. Finally, the combining of similar exposure time periods and delineation of occupational and household agricultural/non-agricultural exposures allowed for assessment of the range of possible external etiological factors involved in breast cancer or endometriosis development. Limitations of the study include those typical of the epidemiological studies combined in meta-analyses such as publication bias, recall bias and exposure misclassification. In addition, EDCs and breast cancer type, along with individual practices of participants, were not distinguished in most studies. There are obvious limitations to this type of bioinformatics analyses. While this analysis generates a hypothesis for potential gene-EDC interactions, further research in a laboratory setting is necessary to validate their role in breast cancer and endometriosis. Although we carefully chose databases, at the time of writing this manuscript, to include comprehensive set of modified genes, we did not assess the entire set of literature on the development of endometriosis and breast cancer. Therefore, possibly we may have missed some potential modified genes in our analysis. Furthermore, epigenetic genes were not included in our analysis that may have excluded other potential gene-EDC interaction pathways leading to breast cancer and endometriosis through these mechanisms. In spite of these limitations, this study presents a clear advantage in the identification of genes with potential of highly probable interactions with EDCs that contribute to the development of breast cancer and endometriosis. Furthermore, generation of gene-EDCs interaction data relevant to breast cancer and endometriosis through this integrative approach provides useful leads for comprehensive understanding of gene-EDCs interaction in the development breast cancer and endometriosis. Research with an integrated bioinformatic, biostatistic and molecular epidemiologic approach is however needed to study the relative contributions of PCB, phthalate and BPA exposure to determine the causality and progression of these complex chronic disease phenotypes in humans.

In summary, the major novel findings of this study are that PCBs exposure may increase risk of breast cancer and endometriosis, in part, as a result of common molecular risk factors. A single exposure to an internal or external environmental factor alone cannot explain the development of a complex chronic disease, such as breast cancer and endometriosis, rather it appears that exposure to multiple environmental and molecular factors across the lifespan and their interactions influence the development of these chronic diseases in an individual. There may be common molecular risk factors between endometriosis and breast cancer. Given the proven contribution of unopposed estrogens to the risk for endometriosis or endometrial neoplasia or breast cancer, it is biologically plausible that an altered endogenous estrogen levels presumably from exposure to estrogen mimicking EDCs may contribute to the risk of these diseases. Our bioinformatics approach helps to identify genes associated with EDCs to generate novel hypothesis to evaluate the relationship between endometriosis and breast cancer. Therefore the present approach to evaluate endocrine disruptor responsiveness and their impacts on the biological systems is consistent with system-wide findings in breast cancer and endometriosis which supports this integrative idea to identify the numerous and complex modes of gene-EDCs interaction in these complex diseases.

## 4. Methods

The resources, workflow, meta-analysis and bioinformatics tools and integration of environmental epidemiologic, genomic and disease databases are shown in Figure 1. The flow chart shows the steps involved in identifying genes that illustrate the link between endometriosis and breast cancer based on environmental response on epidemiologic, genomics, and bioinformatics databases. We used the Comparative Toxicogenomics Database (CTD), Endocrine Disruptor Knowledge Base (EDKB) and KEGG database for assessing estrogenicity of environmental chemicals.

We used EDKB computer-based models to predict affinity for binding of PCBs, Bisphenol A and B and phthalates to the estrogen and androgen nuclear receptor proteins, which revealed the estrogenic potency of each endocrine disruptor.

We mapped environmental chemicals onto the KEGG endocrine disrupting compound, the KEGG pathway and metabolic pathways, particularly synthetic and degradation pathways of EDCs for assessing estrogenic activity.

### 4.1. Data Sources and Searches for Meta-Analysis

A PubMed search was conducted to identify studies of the association between breast cancer or endometriosis and PCBs, phthalates or bisphenol A. We limited our search to studies published in the year 2000 and later, and articles from scholarly publications, including peer review. We identified and screened a total of 125 publications from which we eliminated duplicates, surveys, review articles, animal studies, and letters to the editor. The remaining 59 publications were then reviewed in detail for relevancy to our objective. Title search commands included: PCBs or polychlorinated biphenyls, phthalates, bisphenol A or BPA, organochlorines, and endometriosis or breast cancer.

Study Selection: To be included in our meta-analysis, the study had to meet the following criteria: (1) PCBs, phthalates, or BPA had to be an exposure variable; (2) breast cancer or endometriosis had to be an outcome variable; (3) exposure levels reported in medians, means, geometric means, or TEQs; and (4) estimated odd ratios (ORs) with their 95% confidence intervals (CIs). Exclusion criteria for the initial search were: (1) did not report original results (reviews, comments, letters, *etc.*); (2) results already reported in another study or in a more comprehensive study; (3) geographic studies using GIS, *etc.*; (4) study had less than 4 cases in subgroup of interest; and (5) study did not report timing of exposure.

Meta-analysis was performed and homogeneity was tested by means of the Q statistic [71]. Analysis was completed using Comprehensive Meta-Analysis Version 2.2.046 from Biostat, Inc., which can be downloaded at www.Meta-Analysis.com.

### 4.2. Genomic/Bioinformatic Analyses

We used bioinformatics approach to identify gene-EDCs interactions and diseases association as described previously [72]. Public databases were used for identifying estrogen mimicking endocrine disruptor responsive important genes with complex diseases - breast cancer or endometriosis. We used the following databases:

• The Comparative Toxicogenomics Database (CTD) is located at: http://ctdbase.org/. We searched a list of genes found to be modulated by three selected endocrine disruptors in breast cancer or endometriosis.
• The Environmental Genome Project (EGP) located at: http://www.niehs.nih.gov/research/supported/programs/egp/. All “identified important genes” from the CTD database were included for comparison with the genes in EGP.
• We used The Seattle SNPs database (http://pga.gs.washington.edu) to compare with the genes known to contain variation in breast cancer, endometriosis and exposure to individual EDC.
• The modified genes from the CTD and environmental genome databases curation, were inputted into the GeneVenn program to assess their overlap as depicted in Figure 3.

### 4.3. Literature Based Validation of Genes Showing Links between Endometriosis and Breast Cancer

We investigated to validate genes that were identified using the CTD database that shows the biological plausibility of links between endometriosis and breast cancer. The literature and database search of EDC responsive genes common in both breast cancer and endometriosis lesions collected information on gene cellular localizations and functions, and also published research supporting the genes involvement in the development of both diseases. We conducted the search on on The Human Gene Compendium’s Gene Cards (www.genecards.org), PubMed (www.pubmed.com), the Information Hyperlinked over Proteins (iHOP) Database (www.ihop-net.org), and the Epidemiologic and Bioinformatics Database-http://www.cdc.gov/cancer/npcr/about_inca.htm; http://cancergenome.nih.gov; http://www.cbioportal.org/public-portal/; http://www.endometrialdatabase.com/edb/ databases.

We used Banjo (Duke University, NC) software for probabilistic structure learning of static Bayesian networks using TGCA 2012 breast cancer expression [73]. The goal of this Bayesian analysis was to identify critical gene-gene interactions in breast cancer to validate some of our findings of EDC genomics.

In order to investigate existing literature and ontology based connections between EDC responsive gene lists in breast cancer and endometriosis we also conducted gene enrichment analysis. The same set of genes were used to produce connections that were independent of their established roles in different pathways. This analysis produced gene networks that included EDC responsive genes identified in this study from database and literature searches. According to IPA-defined significance score networks were ordered for Direct and Indirect Relationships, All Data Sources, All Species, and All Tissues and Cell Lines. This public server-based tools allow integration of pathway-related annotations from several public sources including Reactome, KEGG, NCBI Pathway Interaction Database, and Biocarta to interpret interactions among the identified set of genes. By using web-based available tools we produced interactive graphs linking all four EDC responsive gene lists with pathway annotations, allowing for graphical pathway investigation into our gene lists

## Supplementary Materials

Supplementary materials can be found at http://www.mdpi.com/1422-0067/16/10/25285/s1.
