# Supplementary material for: Integrated Bioinformatics, Environmental Epidemiologic and Genomic Approaches to Identify Environmental and Molecular Links between Endometriosis and Breast Cancer
Source: Int J Mol Sci. 2015 Oct 23;16(10):25285–322. doi: 10.3390/ijms161025285 (PMC4632802; doi:10.3390/ijms161025285)
Supplement: Supplementary file 1 [file ijms-16-25285-s001.pdf]

Supplementary Information

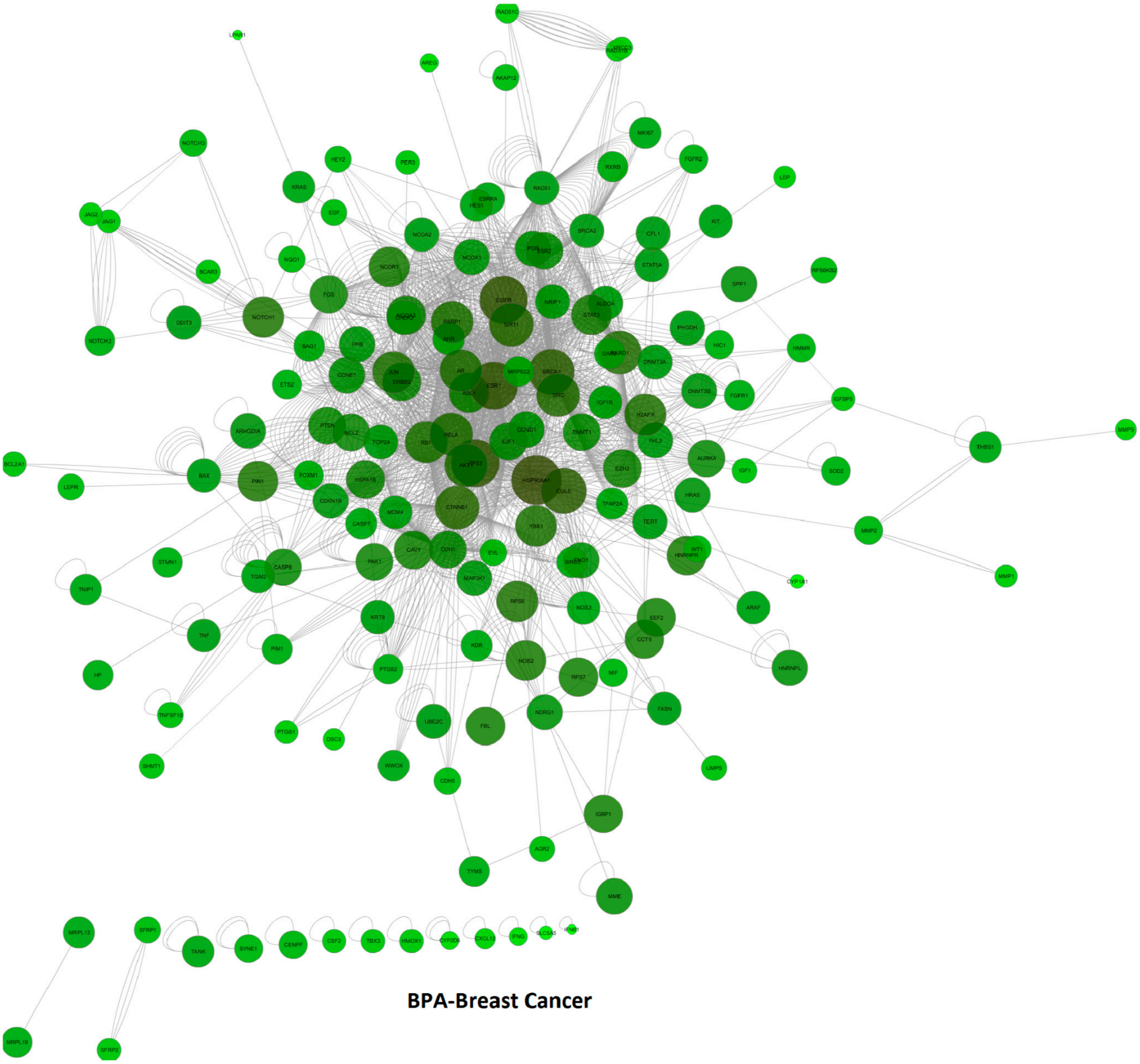

Figure S1. *Cont.*

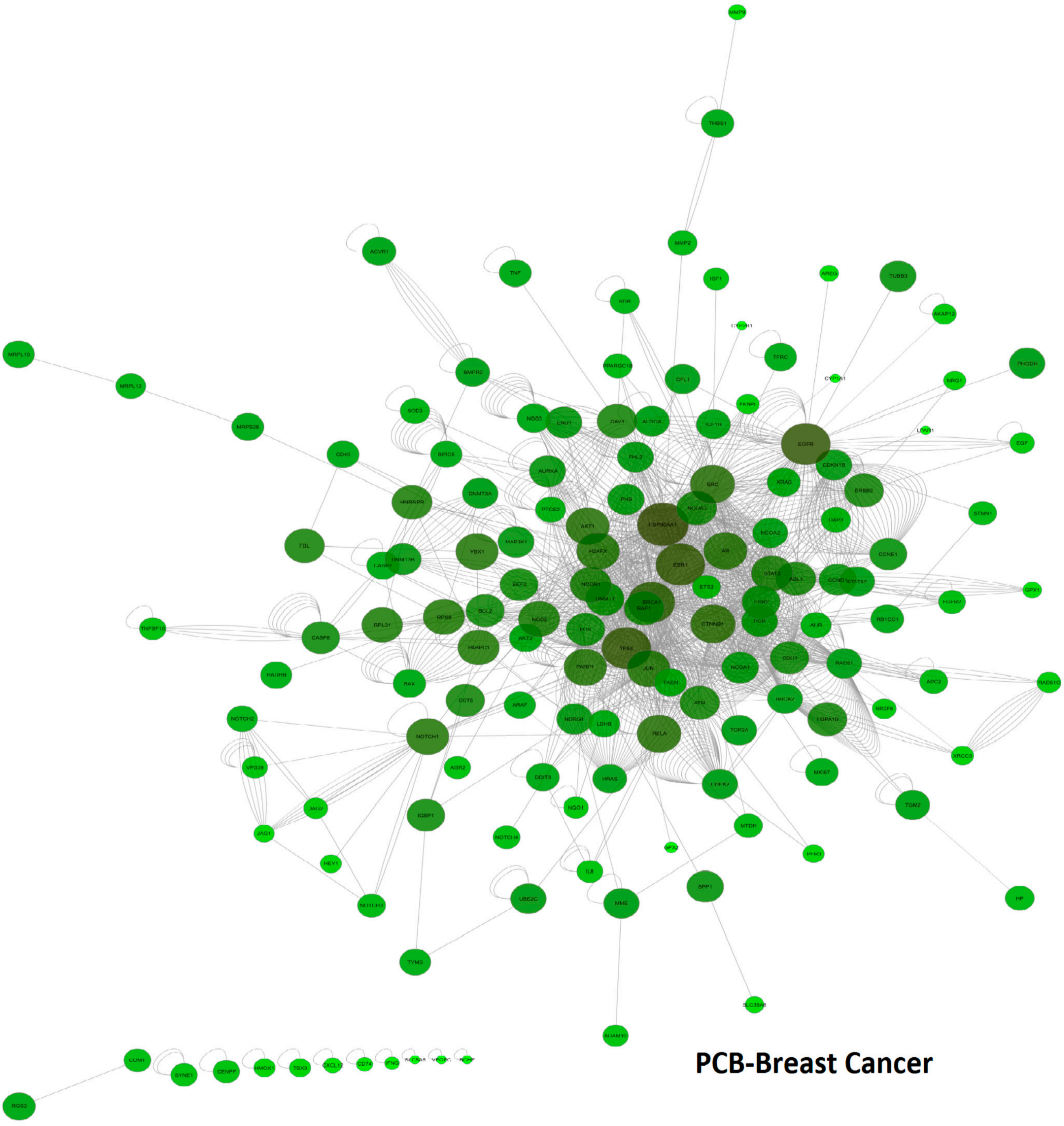

Figure S1. *Cont.*

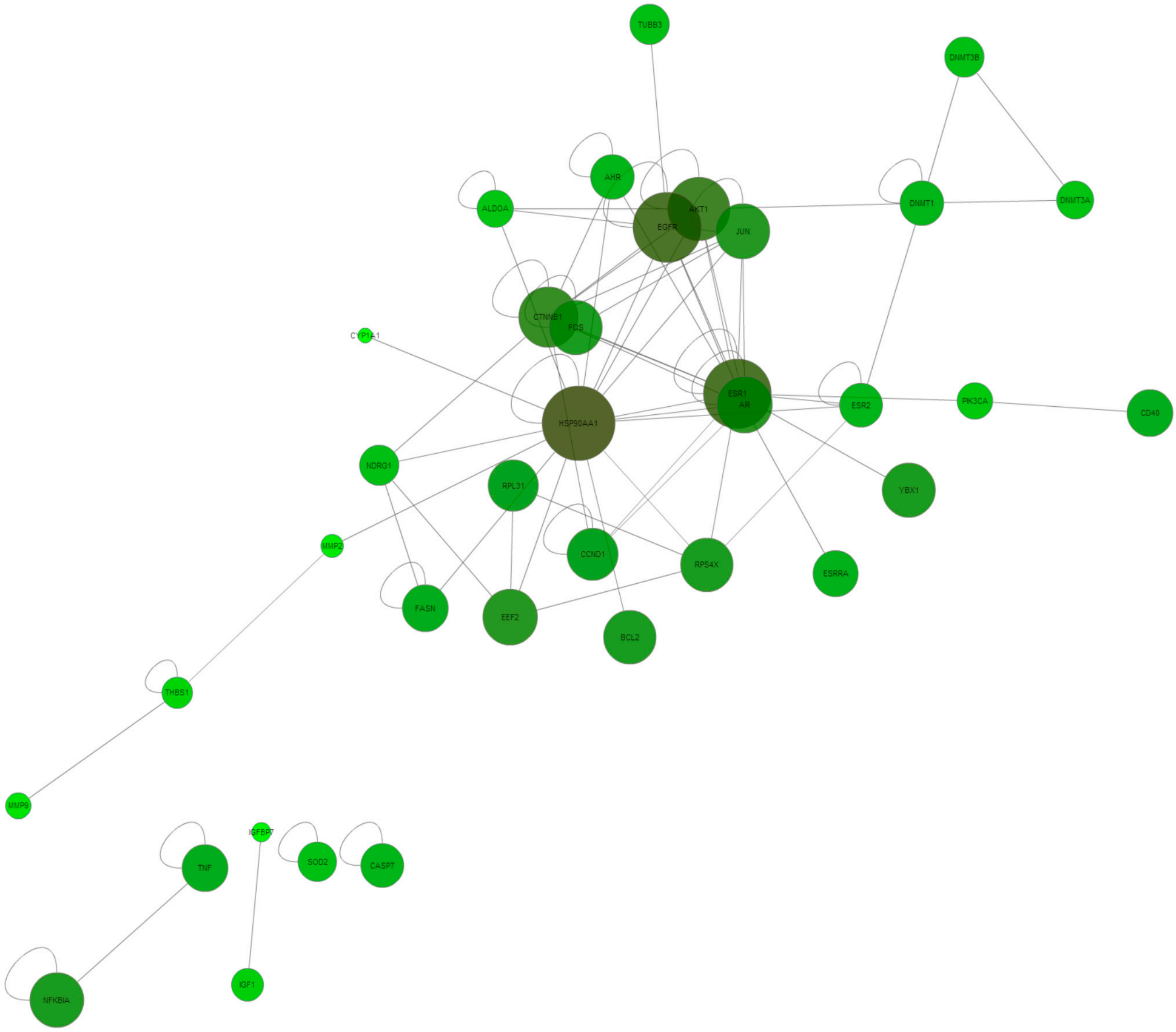

Phthalate-Breast Cancer

Figure S1. Gene Set/Pathway enrichment analysis of genes associated with PCBs, phthalates or bisphenol A in breast neoplasms.

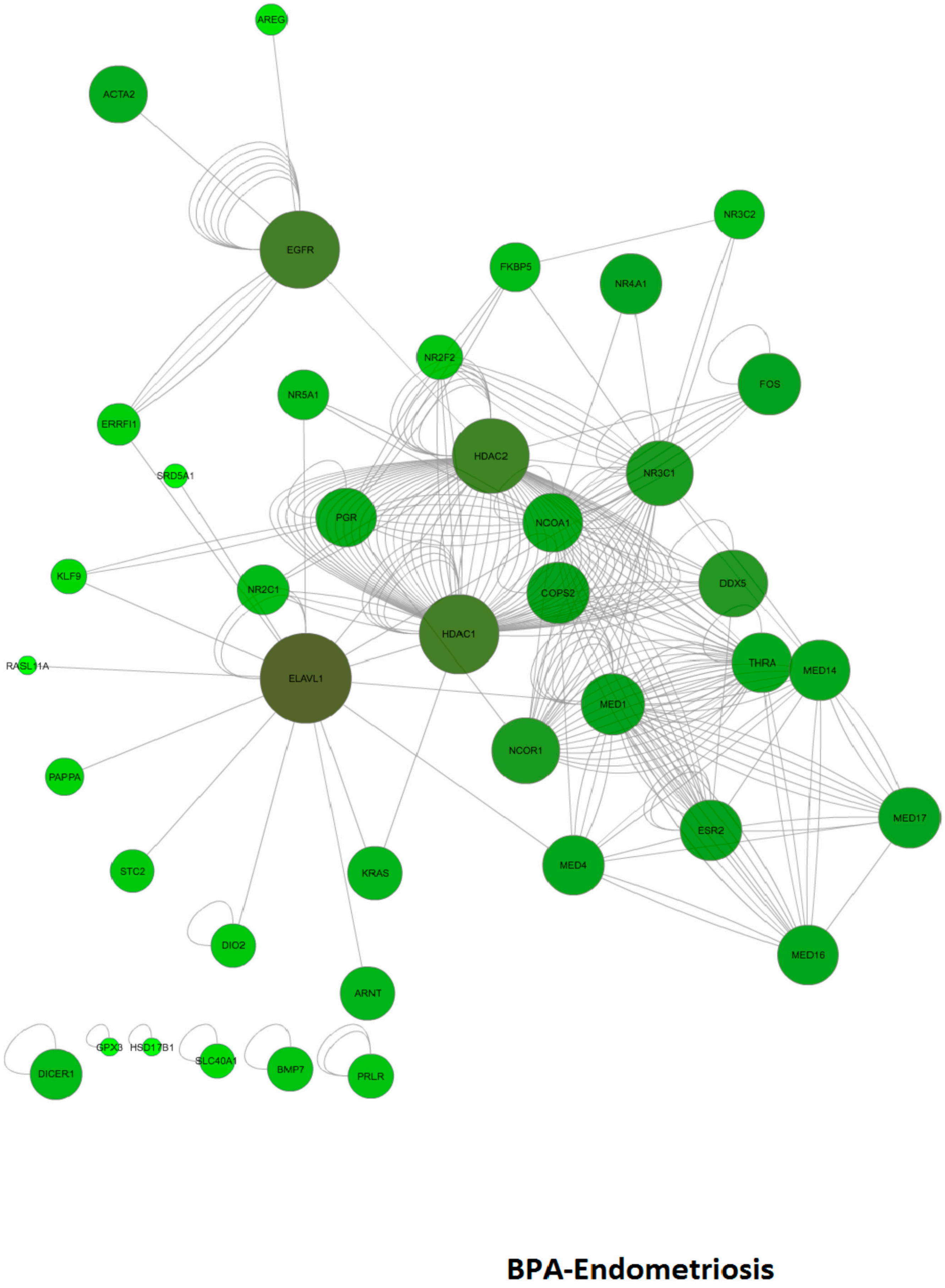

Figure S2. *Cont.*

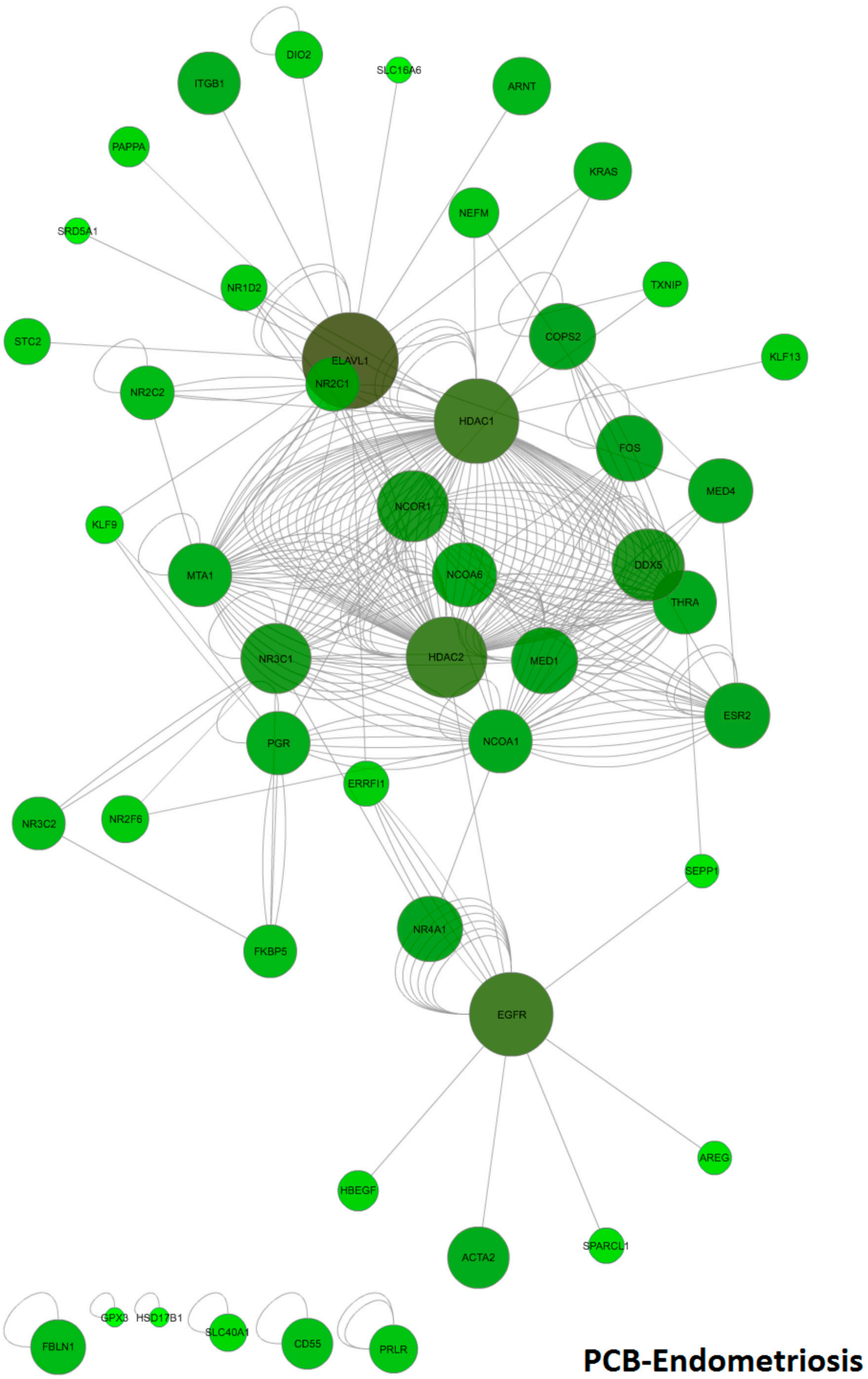

Figure S2. *Cont.*

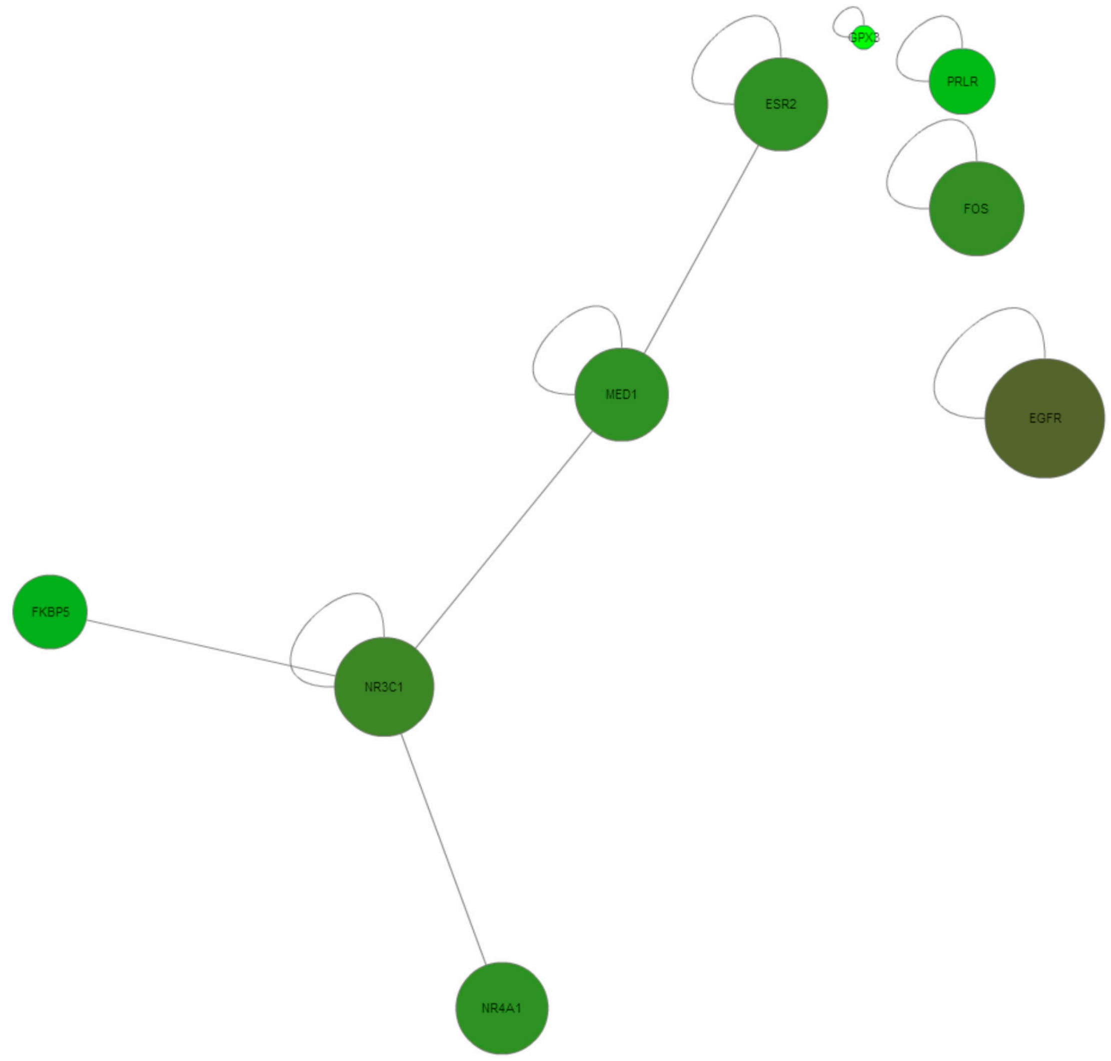

Phthalate-Endometriosis

Figure S2. Gene Set/Pathway enrichment analysis of genes associated with PCBs, phthalates or bisphenol A in endometriosis.
